# Human OTUD6B positively regulates type I IFN antiviral innate immune responses by deubiquitinating and stabilizing IRF3

**DOI:** 10.1128/mbio.00332-23

**Published:** 2023-08-31

**Authors:** Jian Wang, Hui Zheng, Chunsheng Dong, Sidong Xiong

**Affiliations:** 1 Jiangsu Key Laboratory of Infection and Immunity, Institutes of Biology and Medical Sciences, Soochow University, Suzhou, Jiangsu, China; University of Pennsylvania, Philadelphia, Pennsylvania, USA; University of California San Diego, San Diego, California, USA

**Keywords:** antiviral immunity, deubiquitination, OTUD6B, Type I IFN

## Abstract

Elaborate regulation of innate immunity is necessary for the host to effectively respond to invading pathogens. As an important component of antiviral immunity transcription factors, the stability and activity of interferon (IFN) regulatory factor 3 (IRF3) are tightly controlled via multiple post-translational modifications. Here, we identified a human ovarian tumor domain-containing deubiquitinase OTUD6B as a positive regulator of IRF3 that facilitates type I IFN innate antiviral immune signaling. Mechanically, we found that OTUD6B interacts with IRF3 and directly hydrolyzes both the lysine 11 (K11)- and the lysine 33 (K33)-linked polyubiquitin chain, but only K33-linked polyubiquitin at Lys315 of IRF3 is responsible for IRF3 proteasome degradation. Notably, OTUD6B enhanced cellular antiviral responses *in vivo*, as evidenced by mice that overexpressed human OTUD6B were more resistant to RNA virus infection and had reduced viral load and morbidity. These findings revealed a previously unknown role for OTUD6B in the regulation of innate antiviral immunity and may provide a potential target for enhancing host antiviral defense.

**IMPORTANCE:**

Interferon (IFN) regulatory factor (IRF3) is one of the key factors for type I IFN transcription. To sophisticatedly regulate type I IFN antiviral immune response, IRF3 activity is closely controlled by a variety of post-translational modifications. However, the regulatory mechanisms are still not fully elucidated. In the present study, we found that human deubiquitinase OTUD6B positively regulates IRF3-mediated antiviral immune response. OTUD6B can stabilize the IRF3 protein level via hydrolyzing (Lys33)-linked polyubiquitin at Lys315. More importantly, mice with OTUD6B overexpression exhibited more resistance to RNA virus infection. Thus, unlike the previous report that zebrafish OTUD6B negatively regulates the antiviral response by suppressing K63-linked ubiquitination of IRF3 and IRF7, we demonstrate that human OTUD6B actually enhances type I IFN response and has the potential for antiviral therapy.

## INTRODUCTION

The innate immune response is the first line of host defense against viral infection. When host cells encounter viruses, host pattern recognition receptors (PRRs), such as inducible gene I (RIG-I)-like receptors (RLRs), Toll-like receptors (TLRs), and cytosolic dsDNA sensors, can recognize viral nucleic acid (1 – 4). PRRs trigger antiviral signaling and result in the production of type I interferons (IFNs) and proinflammatory cytokines, which are central to efficient host defense against viral infection (1, 3, 5, 6). The RLRs signaling pathway, including RIG-I and MDA5, is primarily responsible for the recognition of cytosolic viral RNAs (3, 7, 8). RLRs recognize viral RNAs through the RNA helicase domain (RLD). Thereafter, the 2CARD tetramer of RLRs acts as a core for initiating mitochondrial antiviral signaling (MAVS) protein polymerization, leading to MAVS prion-like fiber formation. MAVS further recruits tumor necrosis factor receptor-associated factor (TRAF) family proteins to form a MAVS/TRAF3/TRAF6 signalosome. Subsequently, the signalosome induces the activation of serine/threonine-protein kinase (TBK1) and inhibitor of nuclear factor kappa-B kinase subunit epsilon (IKKε), which finally phosphorylates IFN regulatory factor (IRF3). Activated IRF3 translocates to the nucleus for transcription of the antiviral cytokine type I IFN, which eventually leads to type I IFN-mediated antiviral IFN-stimulated gene (ISG) expression (9).

IRF3 is one of the key factors for type I IFN transcription, and its activity is regulated by a variety of post-translational modifications, such as phosphorylation, acetylation, and methylation (10). Among these, MID1, TRIM21, C-CBL, TRIM26, OTUD1, RBCK1, PLPRO, POH1, and RNF5 have been reported to be involved in the ubiquitination of IRF3 (11 – 19). MID1, TRIM21, C-CBL, TRIM26, RBCK1, and RNF5 are E3 ubiquitin ligases that catalyze the formation of IRF3 K48 or K63 ubiquitin chains, whereas OTUD1, PLPRO, and POH1 are deubiquitinating enzymes (DUBs) that cleave IRF3 K48 or K27 ubiquitin chains. These studies have demonstrated that the activity of IRF3 is under exquisite regulation; however, the regulatory mechanisms are still not fully elucidated.

Human ovarian tumor proteases (OTUs), which contain more than 10 family members, are one of the seven families of DUBs and have been identified as key DUBs in regulating the type I IFN innate immune response (15, 20 – 23). However, regarding human OTUD6B, previous studies showed that it is primarily involved in tumorigenesis and regulates cell growth and proliferation (24 – 26). Additionally, it was found that biallelic variants in human OTUD6B cause intellectual disability syndrome (27). The function of OTUD6B in response to viral infection has rarely been reported, except that a recent study found that OTUD6B from zebrafish can negatively regulate the antiviral response by suppressing K63-linked ubiquitination of IRF3 and IRF7 (28). Whether human OTUD6B plays a similar role in viral infections remains elusive.

Here, we performed human OTU screening to systematically examine their antiviral function. We found that contrary to zebrafish OTUD6B, human OTUD6B positively regulates antiviral innate immune responses. The antiviral effects of human OTUD6B are dependent on its deubiquitinating enzymes activity. The OTUD6B interacts with IRF3 and cleaves K11- and K33-linked ubiquitin chains of IRF3. However, the K33-linked, but not the K11-linked, ubiquitin chain on IRF3 is responsible for IRF3 degradation. The IRF3 Lys315 is a major residue for OTUD6B-induced deubiquitination. Furthermore, we found that human OTUD6B enhances type I IFN antiviral immune response in mice upon viral infection. These findings uncover that human OTUD6B can deubiquitinate and stabilize IRF3 and enhance type I IFN antiviral immunity.

## RESULTS

### Screening of the CRISPR-Cas9-mediated OTU knockout library revealed that human OTUD6B executes antiviral functions

**Fig 1:**
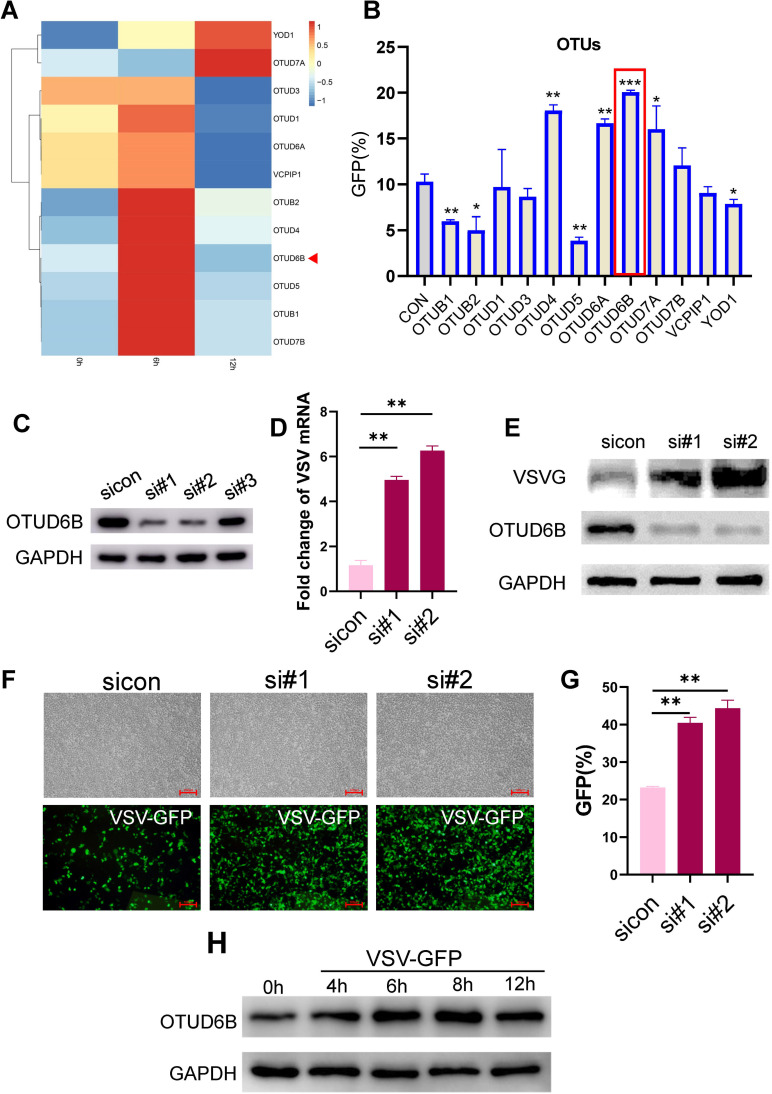
OTUD6B screened in CRISPR-Cas9 knockout (KO) OTUs library exhibited antiviral function. (**A**) The expression level of OTUs in VSV-infected 293T cells was shown in a heat map by color-coded intensity. 293T cells were infected with VSV-GFP (MOI = 1.0) for 6 or 12 h, and then, the mRNA level of OTUs was measured by real-time PCR. (**B**) Viral replication of VSV-GFP infected 293T cells. 293T cells transduced with OTUs knockout lentiviral library and were infected with VSV-GFP for 12 h. Viral infection was measured by flowcytometry. (**C**) 293T cells were transfected with either control siRNA or siOTUD6B. Protein level of OTUD6B was measured by western blot. (**D–G**) 293T cells were transfected with either control siRNA or siOTUD6B. After 36 h post transfection, cells were infected with VSV-GFP for 12 h. The infection was then analyzed by (**D**) real-time PCR, (**E**) western blot, and (**F**) fluorescence microscopy. Scale bars: 100 µm, (**G**) flow cytometry. (**H**) 293T cells were infected with VSV-GFP (MOI = 1.0) at different time points (0, 4, 6, 8, and 12 h). The protein level of OTUD6B was measured by western blot. The data shown are the means ± SD and are the representative of three independent experiments. *P ≤ 0.05; **P ≤ 0.01; ***P ≤ 0.001.

In humans, members of the OTU family have been reported to be involved in antiviral innate immunity (15, 20 – 23, 29, 30). To systematically explore the role of OTU family members in regulating the innate antiviral response, we examined their changes in the expression at different time points upon vesicular stomatitis virus (VSV) infection. We found that the mRNA levels of the OTU family members varied during viral infection (Fig. 1A). Among these, one group containing OTUB1, OTUB2, OTUD4, OTUD5, OTUD6B, and OTUD7B showed obvious upregulation 6 h post-infection, followed by downregulation 12 h post-infection. To further determine the effect of OTU family members on viral replication, we individually downregulated their expression using CRISPR-Cas9 in 293T cells. The downregulated 293T cells were subsequently infected with the VSV-GFP reporter virus, and viral infection was measured using flow cytometry. We found that OTUD4, OTUD6A, OTUD6B, and OTUD7A knockdown promoted VSV replication, whereas OTUB1, OTUB2, OTUD5, and YOD1 knockdown inhibited VSV replication (Fig. 1B). In fact, OTUB1, OTUB2, OTUD5, and OTUD4 screened here have been previously identified by others as having the same effect on viral replication (20, 21, 29), further suggesting the reliability of the screening assay. In the present study, OTUD4, OTUD6A, OTUD6B, and OTUD7A exhibited antiviral effects, with OTUD6B being the most obvious. Alternatively, the expression of endogenous human OTUD6B was downregulated by transfection of specific small-interfering RNA (siRNA) in 293T cells (Fig. 1C), and knockdown of OTUD6B mediated by siRNA consistently promoted viral replication (Fig. 1D through G). We also examined OTUD6B protein levels during viral infection and found that it increased and peaked at 8 h post infection (Fig. 1H), suggesting that OTUD6B may execute an antiviral function during viral infection. When stimulated with IFN, the OTUD6B expression did not change (data not shown), suggesting it may not be an ISG gene.

### Upregulation of OTUD6B inhibited VSV replication

**Fig 2:**
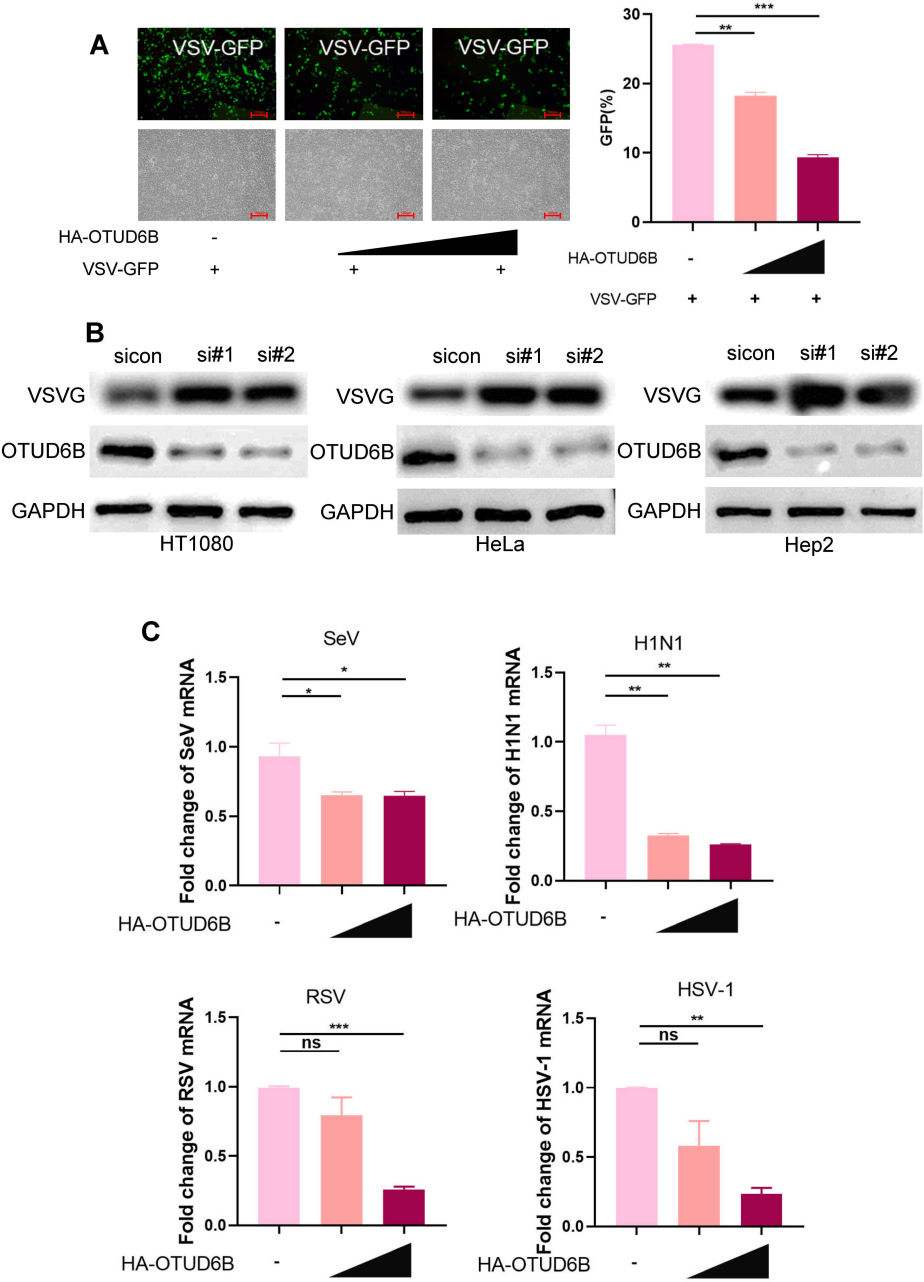
OTUD6B inhibited viral replication. (**A**) 293T cells were transfected with either HA-OTUD6B (1.0 or 1.5 µg) or control vector (1.0 µg). After 36 h post transfection, cells were infected with VSV-GFP (MOI = 1.0) for 12 h. Viral infections were analyzed by fluorescence microscopy and flow cytometry. Scale bars: 100 µm. (**B**) HT1080, HeLa, and Hep2 cells were transfected with OTUD6B siRNAs or control siRNA. After 36 h post transfection, cells were infected with VSV-GFP (MOI = 1.0) for 12 h. Viral infections were analyzed by western blot. (**C**) 293T cells were transfected with HA-OTUD6B (1.0 or 1.5 µg). After 36 h transfection, cells were infected with SeV, H1N1, RSV, and HSV-1 virus (MOI = 1.0) for 12 h. The infections were detected by real-time PCR. The data shown are the means ± SD and are the representative of three independent experiments. *P ≤ 0.05; **P ≤ 0.01; ***P ≤ 0.001; ns, no significance.

To verify the human OTUD6B antiviral effect, we exogenously expressed HA-OTUD6B in 293T cells and infected these cells with VSV 36 h post transfection. Viral replication was measured at 12 h post infection. In contrast to OTUD6B knockdown, we observed that VSV replication was inhibited when OTUD6B was upregulated in 293T cells in a dose-dependent manner (Fig. 2A). Consistent results were observed in other cell lines with OTUD6B downregulation, such as HT1080, HeLa, and Hep2 (Fig. 2B). Furthermore, other RNA viruses including H1N1, SeV, RSV, and the DNA virus HSV-1 were used to investigate the antiviral effect of OTUD6B. Our data showed that OTUD6B overexpression suppressed infection by all these viruses, indicating the broad-spectrum antiviral effects of OTUD6B (Fig. 2C). Collectively, these ﬁndings suggested that OTUD6B can inhibit viral infection.

### OTUD6B promoted type I IFN production in response to viral infection

**Fig 3:**
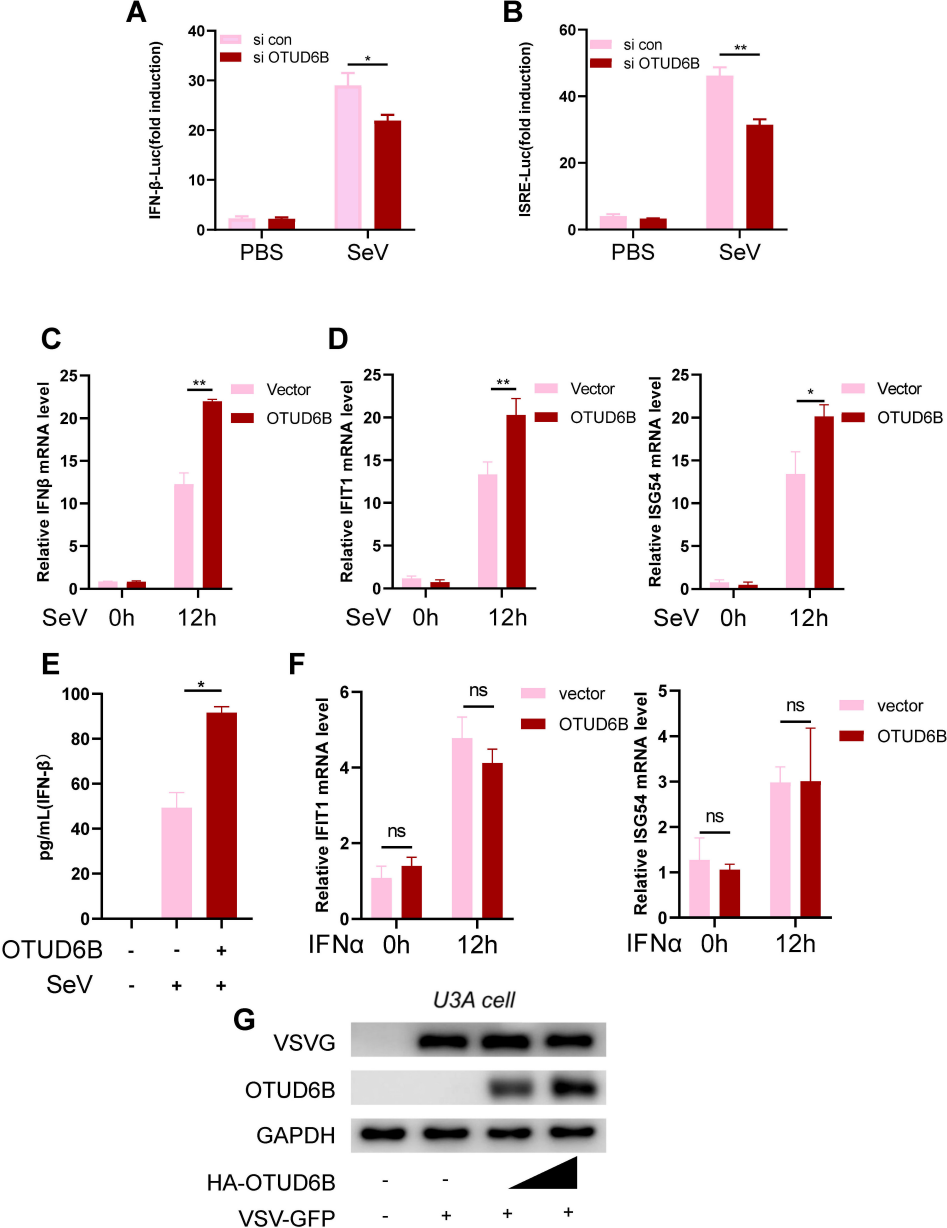
OTUD6B promoted IFN-β production. (**A and B**) 293T cells were transfected with either control siRNA or siOTUD6B, together with IFN-β-Luc (**A**) or IFN-ISRE-Luc (**B**) and Renilla luciferase reporter plasmids for 36 h. The luciferase activity was measured after SeV stimulation for 12 h. (**C and D**) 293T cells were transfected with HA-OTUD6B for 36 h. The IFN-β (**C**) and ISGs (**D**) mRNA levels were detected by real-time PCR after SeV stimulation for 12 h. (**E**) 293T cells were transfected with HA-OTUD6B for 36 h. The secreted IFN-β level in the culture medium was detected by ELISA after 12 h Sev stimulation. (**F**) 293T cells were transfected with HA-OTUD6B for 36 h. Cells were then treated with IFN-α (30 IU/mL) for 15 h. IFN-β and ISGs mRNA levels were detected by real-time PCR. (**G**) STAT1-deficiency cells U3A were transfected with either control or HA-OTUD6B (1.0 or 1.5 µg) plasmid for 36 h. Cells were then infected with VSV-GFP (MOI = 1.0) for 12 h. Viral infections were detected by western blot. The data shown are the means ± SD and are the representative of three independent experiments. *P ≤ 0.05; **P ≤ 0.01; ns, no significance.

The type I IFN immune response is the first defense of the host against the virus. We investigated whether OTUD6B affected type I IFN production or the IFN-mediated signaling pathway. To examine the effect of OTUD6B on type I IFN transcriptional expression, 293T cells were transfected with siOTUD6B and stimulated by SeV for type I IFN production. IFN-β promoter activity was determined using a luciferase reporter assay. Our data showed that OTUD6B knockdown strongly attenuated IFN-β reporter luciferase activity (Fig. 3A) and downstream IFN-stimulatory response element activity (Fig. 3B). This was the same when IFN-β and ISG mRNA levels were detected with OTUD6B overexpression (Fig. 3C and D). In fact, the production of IFN-β, as detected using ELISA, also increased in OTUD6B overexpressed cell culture medium (Fig. 3E). However, when 293T cells were pre-treated with IFNα, there was no difference in the expression of ISGs between the control and OTUD6B overexpression groups, confirming that OTUD6B affects type I IFN production, but not IFN signaling (Fig. 3F). Furthermore, the antiviral effects of OTUD6B disappeared in OTUD6B overexpression and STAT1-deficient U3A cells (Fig. 3G), indicating that the antiviral effects of OTUD6B were dependent on type I IFN signaling. Collectively, these data demonstrated that OTUD6B regulates type I IFN production.

### OTUD6B interacted with IRF3

**Fig 4:**
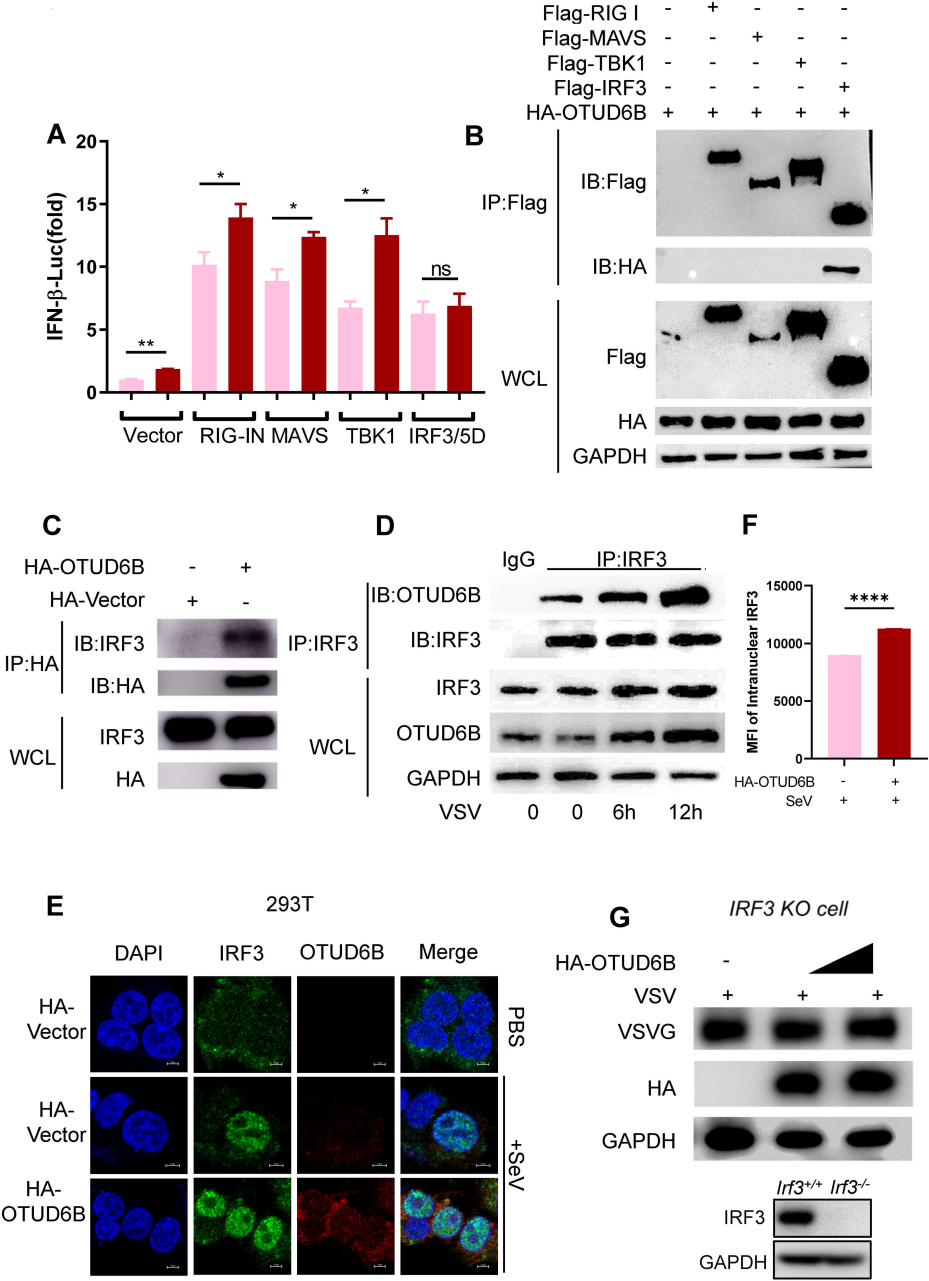
OTUD6B interacted with IRF3. (**A**) 293T cells were transfected with empty vectors (CON) or RIG-IN/MAVS/TBK1/IRF3/5D, together with HA-OTUD6B, IFN-β-luciferase (IFN-β-Luc), and Renilla reporter plasmids. Forty-eight hours after transfection, IFN-β–luciferase activity was analyzed using dual luciferase reporter assay. (**B**) Flag-tagged IRF3 interacted with HA-OTUD6B. 293T cells were transfected with indicated plasmids for 48 h, and the whole cell lysates (WCLs) were immune precipitated with anti-Flag beads. The WCL and immune precipitated proteins were detected using anti-Flag or anti-HA antibodies by western blot. (**C**) HA-tagged OTUD6B interacted with endogenous IRF3. 293T cells were transfected with indicated plasmids for 48 h, and the cell lysates were immunoprecipitated with anti-HA beads. The WCL and immunoprecipitations were detected using indicated antibodies by western blot. (**D**) Endogenous IRF3 and OTUD6B interacted with each other. 293T cells were infected with VSV (MOI = 1) at different time points (0, 6, 12 h), and cell lysates were incubated with IRF3 antibody and then immunoprecipitated with protein A conjugated beads. The immunoprecipitations were detected using anti-IRF3 antibody. (**E**) OTUD6B promotes nuclear IRF3 accumulation in 293T cells. 293T cells were stained by IRF3 and HA antibodies. Cell nuclei were stained by DAPI. The fluorescent images were captured with the Nikon A1 confocal microscope. Scale bars: 5 µM. (**F**) The quantification of the mean fluorescence intensity (MFI) of IRF3 in the nucleus. (**G**) 293T IRF3 KO cells were transfected with either HA-OTUD6B or control vector for 36 h. The cells were infected with VSV-GFP (MOI = 1.0) for 12 h, and viral infections were detected by western blot. The data shown are the means ± SD and are the representative of three independent experiments. *P ≤ 0.05; **P ≤ 0.01; ns, no significance.

RIG-MAVS signaling is an important pathway triggered by cytosolic viral RNAs for host type I IFN production. We explored which key molecules in this signaling pathway were affected by OTUD6B. The 293T cells were co-transfected with one component involved in RIG-MAVS signaling, such as RIG-IN, MAVS, TBK1, and IRF3/5D, together with OTUD6B and IFN-β luciferase reporter (Fig. S1). Plasmid IRF3/5D is a constancy active mutant of IRF3(31). It was shown that IFN-β reporter luciferase activity was no longer enhanced by overexpressing IRF3/5D in the presence of OTUD6B (Fig. 4A), suggesting that OTUD6B might promote type I IFN production via IRF3. Immunoprecipitation experiments confirmed that OTUD6B interacted with IRF3 and not with RIG-I, MAVS, and TBK1 (Fig. 4B), and similar results were obtained using reverse immunoprecipitation (Fig. 4C). Moreover, endogenous OTUD6B and IRF3 constitutively interacted in 293T cells (Fig. 4D). Interestingly, this interaction was enhanced after viral stimulation. Confocal microscopy showed that more IRF3 translocated to the nucleus with overexpressing OTUD6B upon viral infection as measured using mean fluorescence intensity (MFI), the MFI value changed from 8,965 to 11,260 (Fig. 4E and F). Notably, the antiviral effect mediated through OTUD6B upregulation disappeared in IRF3 knockout (KO) 293T cells (Fig. 4G). These findings suggested that OTUD6B positively regulates type I IFN levels by interacting with IRF3.

### OTUD6B stabilized IRF3 protein

**Fig 5:**
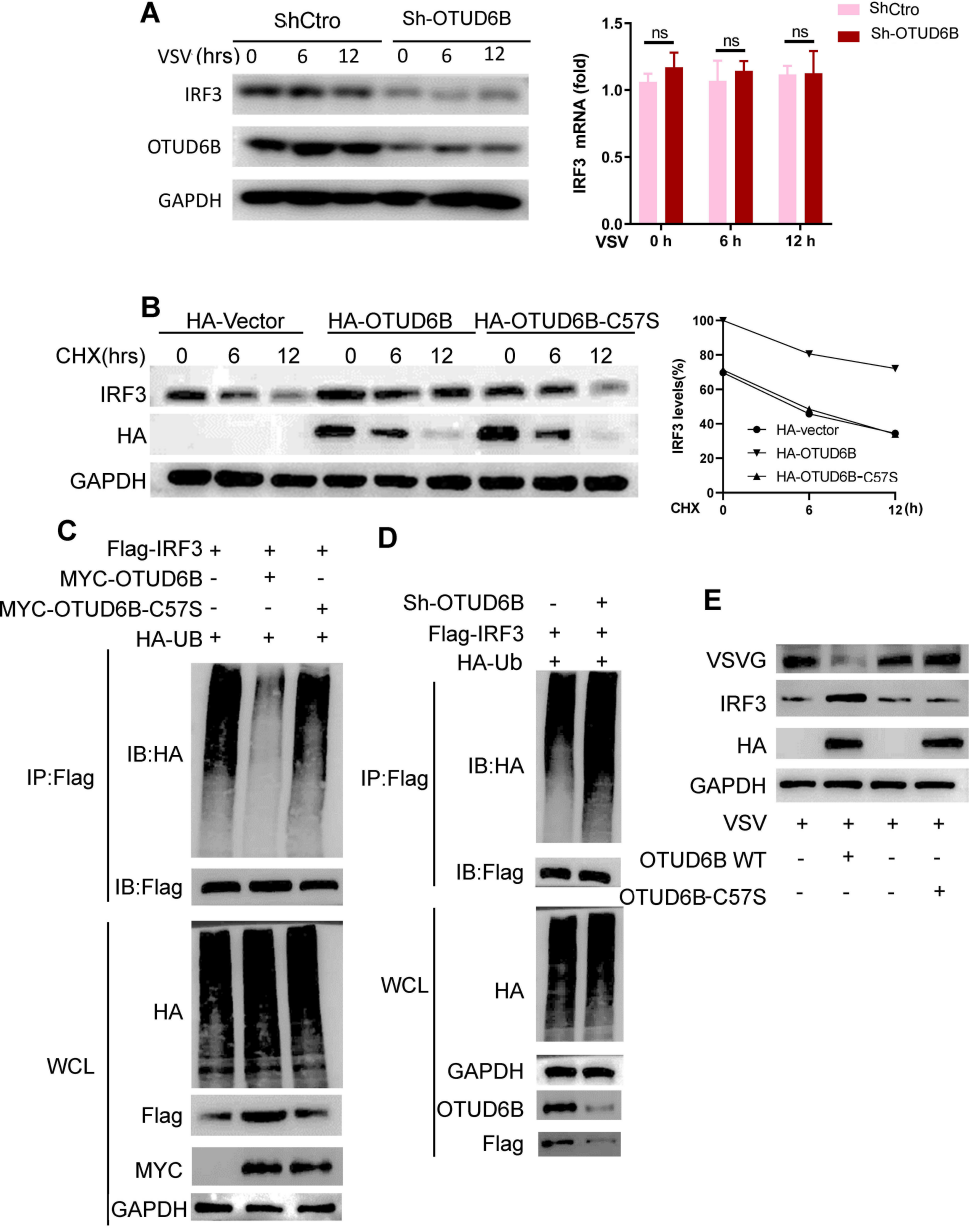
OTUD6B regulated IRF3 protein stability. (**A**) OTUD6B knockdown decreased IRF3 protein level in 293T cells. 293T OTUD6B knockdown cells Sh-OTUD6B and control Vector cells were infected with VSV-GFP (MOI = 1.0) for 0, 6, and 12 h. IRF3 protein levels were measured by western blot (left), and IRF3 mRNA levels were detected by real-time PCR (right). (**B**) OTUD6B increased IRF3 protein stability. 293T cells were transfected with OTUD6B, OTUD6B-C57S, or HA-Vector plasmid, with the treatments of CHX (50 mg/mL) for the indicated times. Cell lysates were analyzed by immunoblotting as indicated. The IRF3 protein level at A time points was calculated as (IRF3_A h/GAPDH_A h)/ (IRF3_0 h/GAPDH_0 h). (**C**) Upregulation OTUD6B reduced polyubiquitination of IRF3. 293T cells were transfected with Flag-IRF3, together with HA-Ub, and MYC-OTUD6B or MYC-OTUD6B-C57S. Immunoprecipitation was performed using anti-Flag beads, followed by immunoblotting using an anti-HA antibody. (**D**) Downregulation OTUD6B increased polyubiquitination of IRF3. 293T Sh-OTUD6B cells were transfected with Flag-IRF3, together with HA-Ub. Immunoprecipitation was performed using anti-Flag magnetic beads, followed by immunoblotting using an anti-HA antibody. (**E**) The antiviral effects of OTUD6B were deubiquitinase activity dependent. 293T cells were transfected with either vector, HA-OTUD6B or HA-OTU6B-C57S, for 36 h. Cells were infected with VSV-GFP (MOI = 1.0) for 12 h. Viral infections were analyzed by western blot. The data shown are the means ± SD and are the representative of three independent experiments. *P ≤ 0.05; **P ≤ 0.01; ns, no significance.

OTUD6B has been reported to be a deubiquitinase (27). Given that OTUD6B interacts with IRF3, an important type I IFN transcription factor, we speculated that OTUD6B may regulate IRF3 protein levels through deubiquitination. OTUD6B knockdown strongly downregulated cellular IRF3 protein levels but not IRF3 mRNA level (Fig. 5A). To address how OTUD6B regulates IRF3 protein levels, we traced IRF3 stability by using the protein synthesis inhibitor cycloheximide (CHX) with OTUD6B overexpression. OTUD6B mutant OTUD6B-C57S was generated as a catalytic activity-defective mutant. Endogenous IRF3 protein levels were higher in OTUD6B overexpressing 293T cells at all time points after CHX treatment, and the degradation rate was slower compared with that of vector or C57S mutant transfected cells (Fig. 5B). We further investigated whether OTUD6B regulates IRF3 protein ubiquitination levels. Our results showed that overexpression of OTUD6B, and not defective OTUD6B-C57S, dramatically attenuated the ubiquitination levels of exogenously expressed IRF3, demonstrating that OTUD6B does reduce IRF3 ubiquitination levels and its deubiquitinase activity is critical (Fig. 5C). In contrast, OTUD6B knockdown enhanced IRF3 ubiquitination modification levels (Fig. 5D). In addition, we found that when the catalyzing activity site of OTUD6B was mutated, it was no longer able to exert an antiviral effect compared to the original OTUD6B (Fig. 5E). These findings suggested that OTUD6B stabilizes IRF3 in a deubiquitinating enzyme activity-dependent manner.

### OTUD6B decreased the K33-linked ubiquitination of IRF3

**Fig 6:**
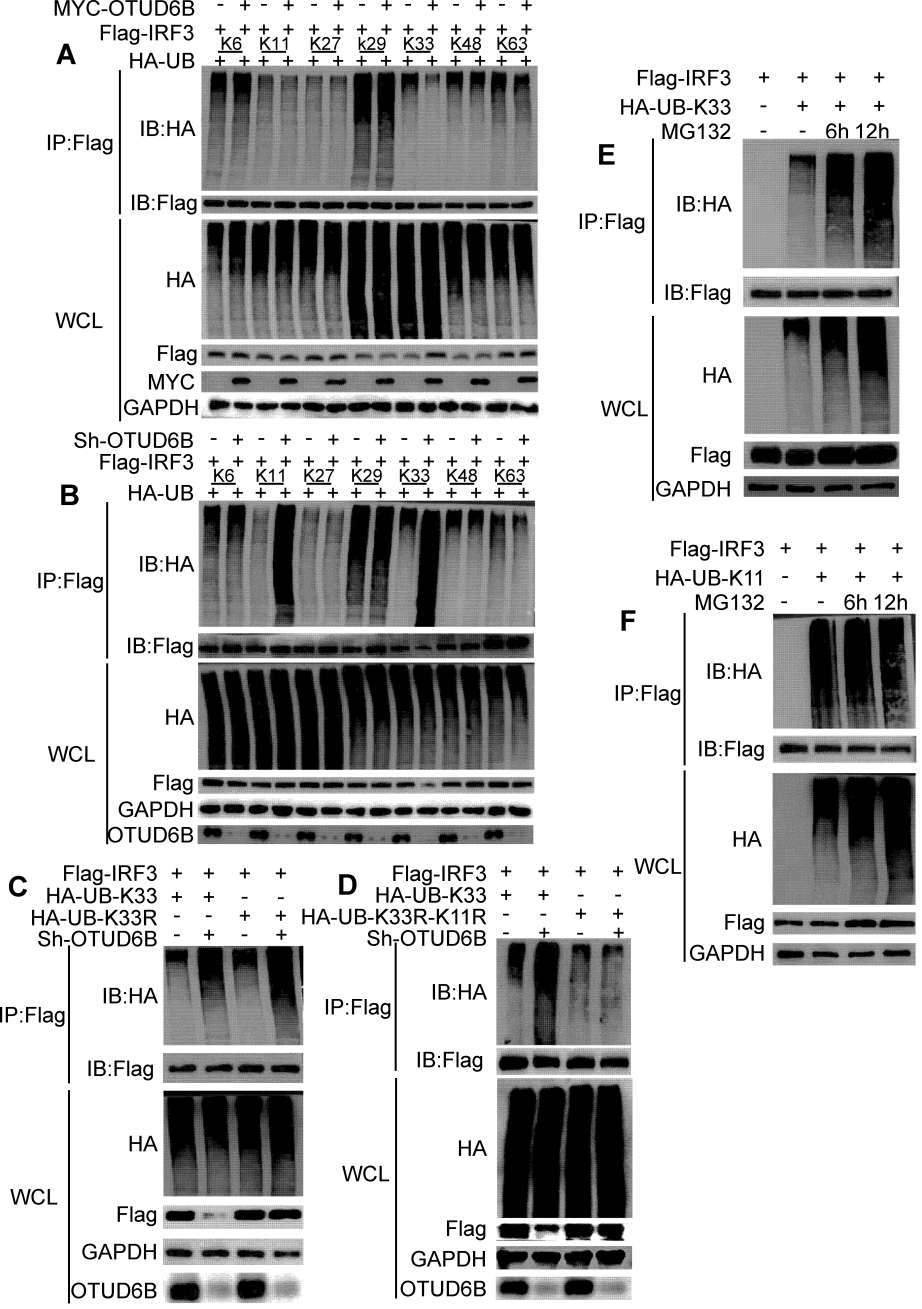
OTUD6B decreased the K33-linked ubiquitination of IRF3. (**A and B**) 293T cells were transfected with Flag-IRF3, together with HA-Ub-K6, HA-Ub-K11, HA-Ub-K27, HA-Ub-K29, HA-Ub-K33, HA-Ub-K48, or HA-Ub-K63, and MYC-OTUD6B or with Sh-OTUD6B knockdown. Immunoprecipitation was performed using anti-Flag magnetic beads, followed by immunoblotting using an anti-HA antibody. (**A**) OTUD6B upregulation reduced K33-linked ubiquitination, while (**B**) OTUD6B downregulation enhanced K11- and K33-linked ubiquitination in 293T cells. (**C**) OTUD6B catalyzed the K33-linked ubiquitination. 293T OTUD6B knockdown cells and its control were transfected with Flag-IRF3, together with HA-UB-K33 or HA-UB-K33R. Immunoprecipitation was performed using anti-Flag magnetic beads, followed by immunoblotting using an anti-HA antibody or an anti-Flag antibody. (**D**) OTUD6B catalyzed the K33- and K11-linked IRF3 ubiquitination. 293T OTUD6B knockdown cells and its control were transfected with Flag-IRF3, together with HA-UB-K33 or HA-UB-K33R-K11R. Immunoprecipitation was performed using anti-Flag magnetic beads, followed by immunoblotting using an anti-HA antibody or an anti-Flag antibody. (**E and F**) 293T cells seeded in the 60-mm dish were transfected with indicated plasmids for 36 h with MG132 (10 mM) treatments. The cell lysates were incubated with anti-Flag magnetic beads, and (**C**) K33- or (**D**) K11-linked ubiquitination level was analyzed by western blot. The data shown are the means ± SD and are the representative of three independent experiments. *P ≤ 0.05; **P ≤ 0.01; ns, no significance.

K48- and K63-linked ubiquitination are the most studied ubiquitination modifications. To investigate the type of ubiquitin chain that IRF3 OTUD6B cleaves, we analyzed the type(s) of IRF3 polyubiquitination mediated by OTUD6B. To this end, HA-Ub-K6, HA-Ub-K11, HA-Ub-K27, HA-Ub-K29, HA-Ub-K33, HA-Ub-K48, and HA-Ub-K63 were transfected into 293T cells with OTUD6B overexpression or knockdown. The results showed that overexpression of OTUD6B largely downregulated K33-linked ubiquitination of IRF3, but not other types of polyubiquitination (Fig. 6A), which was consistent with changes in endogenous IRF3 K33-linked ubiquitination (Fig. S2). However, in addition to K33-linked ubiquitination, OTUD6B knockdown also marginally upregulated K11-linked ubiquitination (Fig. 6B). The K33R and K33R-K11R ubiquitin mutant experiments further demonstrated that OTUD6B did catalyze the K11- or K33-linked ubiquitination of IRF3 (Fig. 6C and D). To investigate which kind of ubiquitination that OTUD6B mediates was associated with IRF3 degradation, 293T cells were transfected with the HA-K33 or HA-K11 plasmid together with the Flag-IRF3 plasmid for 36 h. The transfected cells were treated with MG132, and K33- or K11-linked ubiquitination was examined at 6 and 12 h after MG132 treatment. We found that the K33-linked, and not K11-linked, ubiquitination level of IRF3 was greatly upregulated using MG132 treatment, suggesting that K33-linked IRF3 ubiquitination may be associated with proteasome-dependent IRF3 degradation (Fig. 6E and F). When the cells were treated with CHX, the endogenous IRF3 protein level was significantly decreased in HA-K33 transfected group compared to the control group, while there was no change in the HA-K11 transfected group (Fig. S3). These findings indicated that OTUD6B target both K11- and K33-linked polyubiquitination of IRF3, but the ability to stabilize its protein level is attributed to K33-linked polyubiquitin cleavage.

### OTUD6B decreased IRF3 ubiquitination at the Lys315 residue

**Fig 7:**
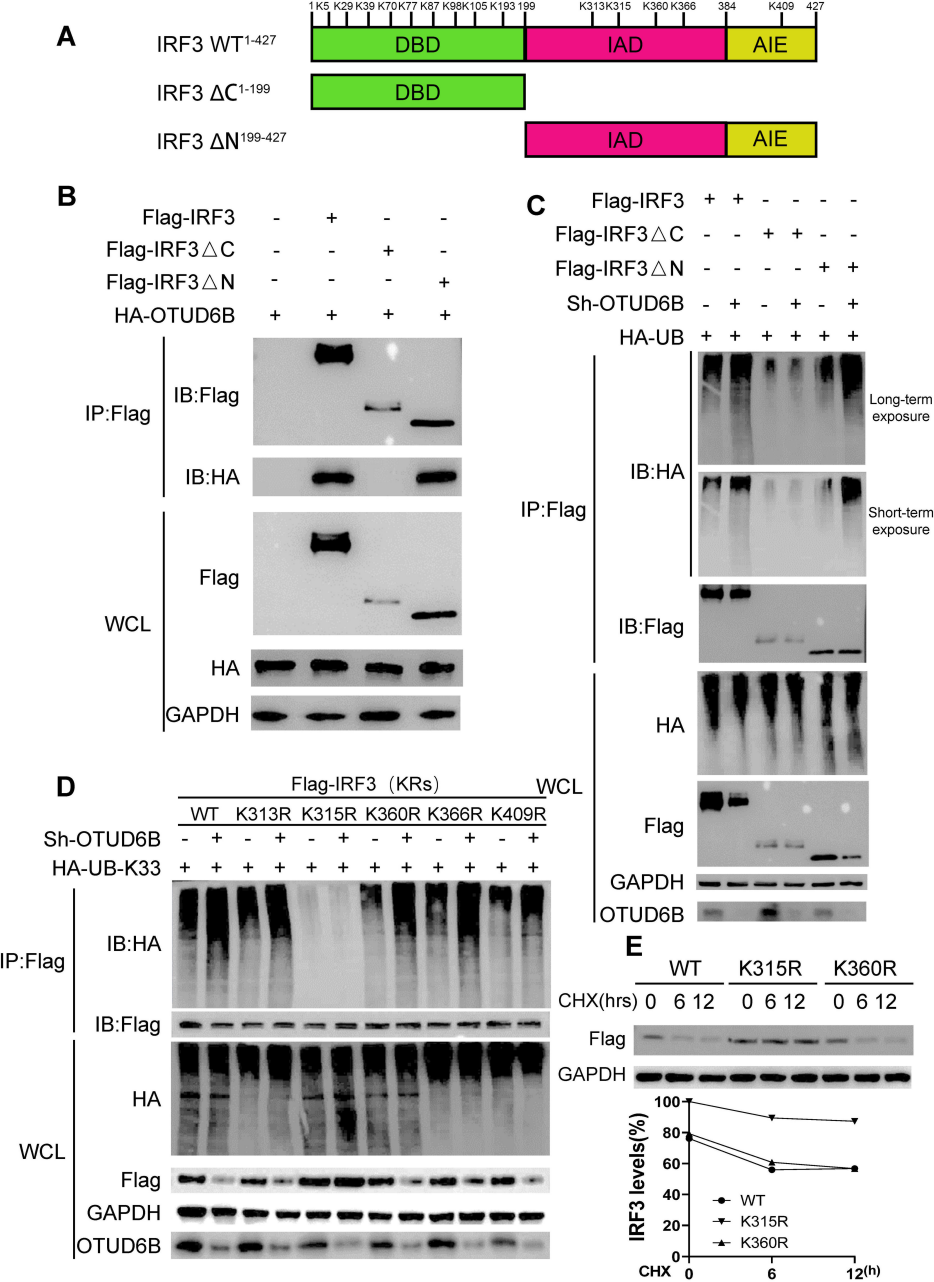
Lys315 is a major lysine residue of IRF3 for deubiquitination induced by OTUD6B. (**A**) Schematics of IRF3ΔC^1–199^ and IRF3ΔN^199–427^ constructs. (**B**) OTUD6B interacted with IRF3 C-terminal. 293T cells were transfected with indicated plasmids for 36 h, and cell lysates were immunoprecipitated with anti-Flag beads followed by immunoblotting. (**C**) OTUD6B catalyzed IRF3 C-terminal deubiquitination. 293T cells were transfected with indicated plasmids, and immunoprecipitation was performed using anti-Flag magnetic beads, followed by immunoblotting using an anti-HA antibody. (**D**) OTUD6B catalyzed IRF3 deubiquitination at Lys315 residue. 293T cells were transfected with indicated plasmids. Immunoprecipitation was performed using anti-Flag magnetic beads, followed by immunoblotting using an anti-HA antibody. (**E**) IRF3 K315R was more stable than IRF3 wild type. 293T cells were transfected with either Flag-IRF3 (WT) or mutant (K315R) or mutant (K360R) for 24 h, with the treatments of CHX (50 mg/mL) for the indicated times. IRF3 protein levels were measured by western blot. The IRF3 protein levels at A time points were calculated as (IRF3_A h/GAPDH_A h)/(IRF3_0 h/GAPDH_0 h). The data shown are the means ± SD and are the representative of three independent experiments. *P ≤ 0.05; **P ≤ 0.01; ns, no significance.

To elucidate the effect of OTUD6B on IRF3 ubiquitination levels, we identified the potential ubiquitination residue(s) catalyzed by OTUD6B. The human IRF3 protein has three conserved domains and consists of 427 amino acids, with 14 lysine residues. Thus, we first split IRF3 into two truncated mutants, IRF3-ΔN and IRF3-ΔC, in which the N- or C-terminal domain was deleted (Fig. 7A). Through immunoprecipitation experiments, we found that OTUD6B interacted with the IRF3 C-terminal domain (IRF3-ΔN), and not with the N-terminal domain (IRF3-ΔC), suggesting that OTUD6B catalyzing site(s) are distributed in the C-terminal of IRF3 (Fig. 7B). The *in vitro* deubiquitination assay confirmed that overexpressing OTUD6B in 293T cells reduced the IRF3-ΔN ubiquitination level, which was in line with the result of full-length IRF3 (Fig. 7C). IRF3-ΔN includes IRF3-IAD and IRF3-AIE domains (10), and the main ubiquitin receptor residues in both domains are unclear. Therefore, we individually mutated all five lysine residues, including Lys313, Lys315, Lys360, Lys366, and Lys409 in the IAD and AIE domains. We found that four of these IRF3 mutants, IRF3-K313R, IRF3-K360R, IRF3-K366R, and IRF3-K409R, still showed upregulated ubiquitination, which was comparable to that of full-length IRF3 in OTUD6B knockdown cells. However, this difference disappeared in K315R mutated IRF3, suggesting that Lys315 in IRF3 may be a major ubiquitin acceptor residue that is cleaved by OTUD6B (Fig. 7D). Furthermore, wild-type IRF3 and IRF3-K315R plasmids were transfected into OTUD6B knockdown cells. CHX assay-indicated IRF3 was more stable with K315R mutation compared with that of wild-type IRF3 (Fig. 7E). More importantly, the viral replication as measured by VSV G protein reduced in K315R mutant transfected cells, suggesting that lys315 is a key amino site for OTUD6B-mediated enhancement of antiviral effects (Fig. S4). Collectively, these findings demonstrated that OTUD6B inhibits IRF3 ubiquitination at Lys315.

### OTUD6B promoted the type I IFN antiviral immune response *in vivo*

**Fig 8:**
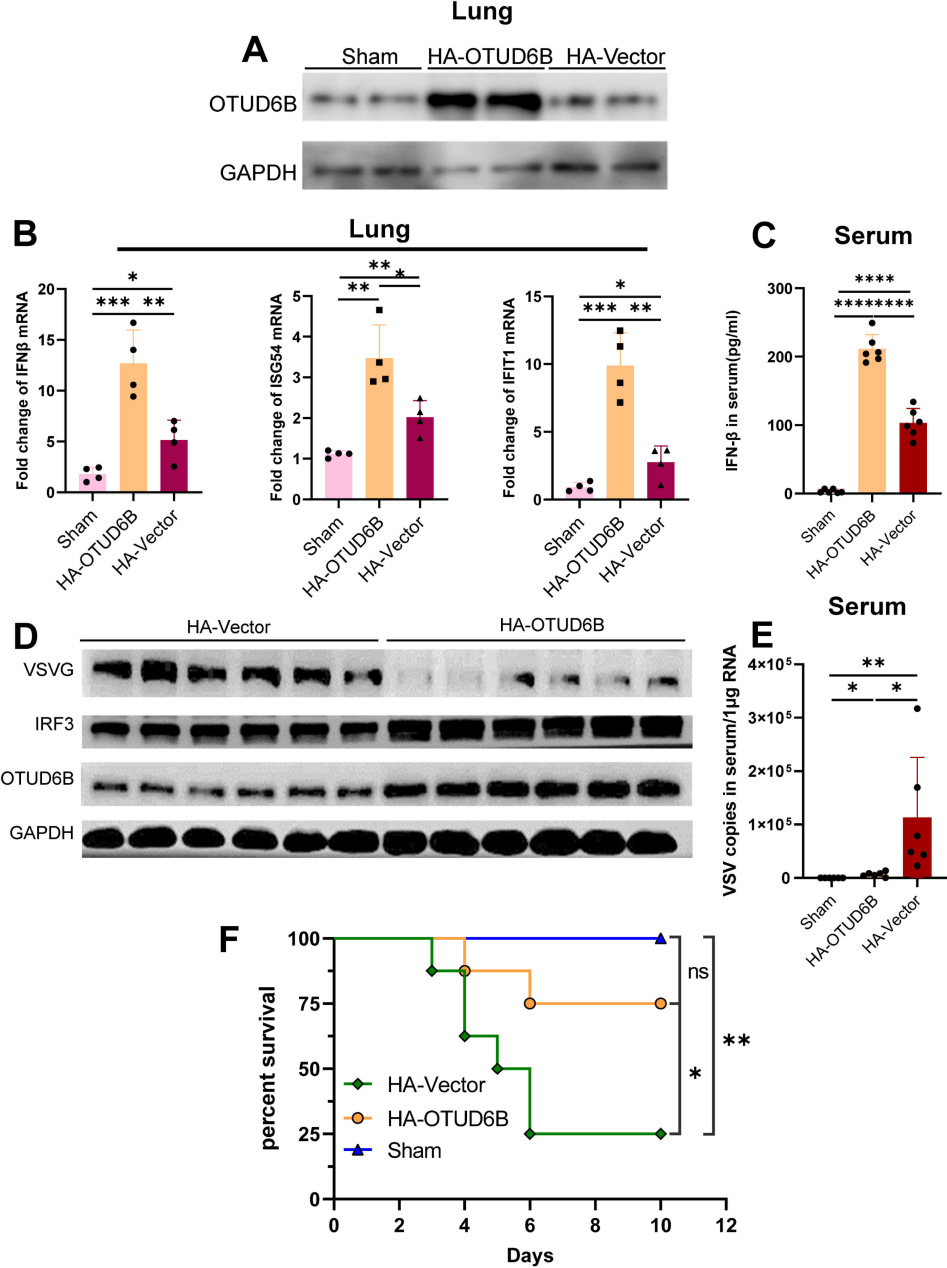
OTUD6B protected mice from viral infection *in vivo*. (**A**) The C57BL/6 mice were i.v. injected with PEI-packaged OTUD6B overexpression plasmid (HA-OTUD6B), control HA-CMV (Vector), and PBS (Sham). OTUD6B expression of mice lung tissues was detected by western blotting on day 2 post injection. (**B and C**) On day 1 post injection, the mice were intranasally infected with 1 × 10^8^ PFU VSV-GFP. The mRNA levels of IFN-β and ISGs in mice lung tissues were analyzed by (**B**) real-time PCR, and (**C**) serum IFN-β level was detected by ELISA on day 3 post infection. (**D and E**) On day 1 post injection, the mice were intranasally infected with 1 × 10^8^ PFU VSV-GFP. The protein levels of mouse IFN-β in sera were analyzed by (**D**) ELISA, and (**E**) serum viral titer was detected by quantitative PCR on day 3 post infection. (**D**) Survival analysis of OTUD6B-upregulated mice. Six- to 8-week-old mice (*n* = 8/group) were intranasally infected with 1 × 10^8^ PFU VSV-GFP, and mortality was observed for 10 days. The survival rate was analyzed using Graphpad prism Kaplan–Meier curve method. The data shown are the means ± SD and are the representative of three independent experiments. *P ≤ 0.05; **P ≤ 0.01; ***P ≤ 0.001; ****P ≤ 0.0001; ns, no significance.

Homozygous OTUD6B knockout mice die at birth, are smaller in size, and have congenital heart defects (27). To investigate the role and functional importance of OTUD6B in the host antiviral response *in vivo*, we upregulated human OTUD6B expression in mice using intravenous injection of polyetherimide (PEI)-packaged plasmids. Exogenous OTUD6B expression was detected in mouse lung tissue 2 days post injection (Fig. 8A). Thereafter, we intranasally infected OTUD6B-upregulated mice with VSV and found that IFN-β production was significantly increased in the lung tissue (Fig. 8B) and serum (Fig. 8C) of OTUD6B-upregulated mice compared with that in control vector-injected mice. Consistently, ISG expression was higher in OTUD6B-upregulated mice (Fig. 8B), and less viral infection was detected in their lung tissue (Fig. 8D) and serum (Fig. 8E) than in control mice 3 days post infection. When viral infection was extended to 10 days, survival results showed that OTUD6B-upregulated mice were more resistant to VSV infection than control vector-injected mice (Fig. 8F). Collectively, our data suggested that OTUD6B-upregulated mice possess a more potent type I IFN antiviral defense against VSV infection.

## DISCUSSION

**Fig 9:**
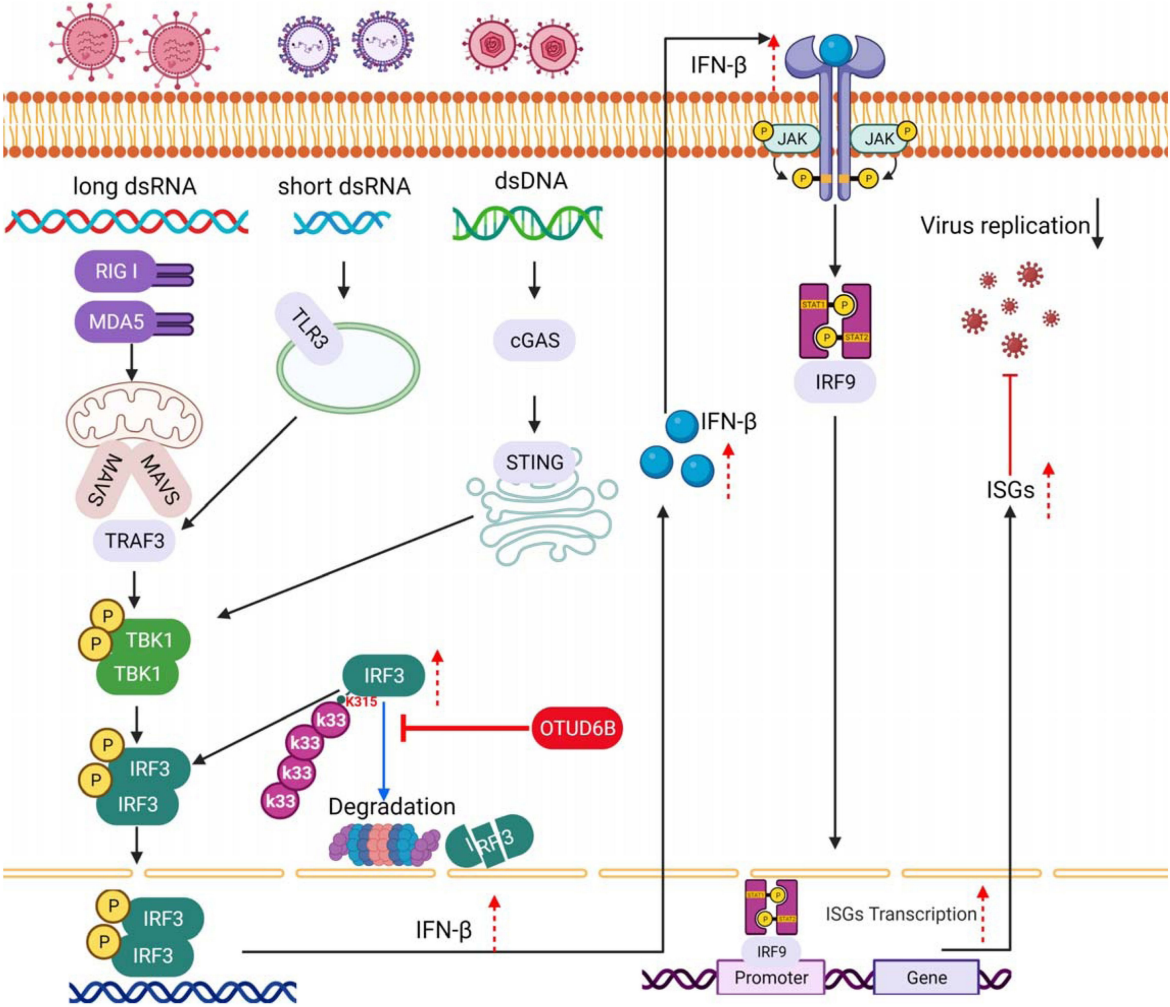
Proposed model for regulation of IRF3 protein levels via OTUD6B. OTUD6B interacts with cytoplasmic IRF3, cleaves its K33-linked ubiquitin chains, and inhibits its proteasomal degradation. Therefore, OTUD6B sustains IRF3 levels in the nucleus and enhances type I IFN transcription and the subsequent downstream ISG expression for antiviral functions.

Type I IFN is induced during the host antiviral innate immune response and is responsible for inhibiting virus replication, clearing virus-infected cells, and facilitating adaptive immune responses. This process is sophisticatedly regulated at distinct levels to ensure proper production of type I IFN against viral infection because uncontrolled or excessive immune responses cause pathological immunity and autoimmune diseases in the host (31 – 33). IRF3 is a key transcription factor involved in innate immune signaling. Functional regulation of IRF3 via post-translational modifications, including phosphorylation, ubiquitination, SUMOylation, acetylation, ISGylation, and glutathione peptidation and methylation, has profound biological and physiological effects on antiviral and autoimmune diseases (15, 34). The number of reported proteins regulating IRF3 activity is increasing as research progresses (10). Although a recent study showed that zebrafish OTUD6B negatively regulates RNA virus-triggered innate immunity by deubiquitinating IRF3 and IRF7 via K63-linked ubiquitination (28), we identified the mammalian human deubiquitinating enzyme OTUD6B that positively regulates antiviral type I IFN response by promoting IRF3 stability, which is different from a previous study. This may be due to differences in OTUD6B species, and the amino acid identity between humans and zebrafish is only 64%. The proposed model for the regulation of IRF3 protein levels via OTUD6B is shown in Fig. 9.

DUBs are ubiquitin hydrolases that cleave mono- or chained-ubiquitin from their substrates. The roles of IRF3 K48- and K63-linked ubiquitination modifications have been extensively reported (11 – 14). A series of DUBs have been identified to modulate innate immune responses by regulating atypical ubiquitination (K0, K6, K11, K27, K29, or K33) of innate immune signaling (35). In fact, the atypical ubiquitination modification of IRF3 plays an important role, which is crucial for its stability and activity. Recent studies have shown that OTUD1 negatively regulates the activity of IRF3 by hydrolyzing its K6-linked ubiquitin chain and attenuating its DNA-binding ability of IRF3 (15). In addition, PSMD14 was reported to promote protein stabilization by cleaving the K313 K27-linked ubiquitin chain of IRF3 (18), which is consistent with zebrafish FOXO3 degrading the protein levels of IRF3 by promoting K27-linked polyubiquitination of IRF3 (36). However, whether there is a DUB that regulates K33-ubiquitination of IRF3 remains unclear. Herein, we identified OTUD6B as a deubiquitinating enzyme that cleaves the IRF3 both K11- and K33-linked ubiquitin chain, but only K33-linked ubiquitination is important for IRF3 stability. Indeed, it was confirmed using anti-K33 ubiquitin antibody, and knockdown OTUD6B obviously increased the K33-linked ubiquitination level of IRF3 (Fig. S2). This is in line with the report that glycine decarboxylase (GLDC)-linked K33 ubiquitination increases its proteasome-dependent degradation (37), and K11-linked ubiquitination in IRF3 does not seem to be associated with proteasomal degradation according to our MG132 treatment. It would be interesting to further explore the significance of K11-linked ubiquitination on IRF3 function in the future.

As an atypical ubiquitination, K33-linked ubiquitination was first reported to be associated with T cell activation (38, 39). The E3 ligase Nrdp1 increases ubiquitination of the Lys33 linkage of Zap70 and promotes its dephosphorylation by acid phosphatase-like proteins Sts1 and Sts2, thereby terminating early TCR signaling in CD8(+) T cells (38). In another case, in mice deficient in E3 ligases Cbl-b and Itch, double-mutant T cells exhibit increased polyubiquitination of the TCR-zeta K33 linkage, which enhances their phosphorylation and Zap-70 binding, leading to enhanced T cell activation (39). Subsequent studies have revealed that K33-ubiquitination is associated with DNA damage and repair (40, 41), protein translocation (42), cellular autophagy (43, 44), and tumorigenesis with glycine metabolism (37). A recent study showed that Avibirnavirus VP3 protein significantly inhibited the TRAF3 Lys155 position K33 ubiquitin linkage, blocked the formation of the TRAF3-TBK1 complex, and thus attenuated the MDA5-mediated type I IFN signaling pathway (45). Furthermore, the ubiquitin ligase NF-X1 catalyzes GLDC K33-linked ubiquitination at Lys 544 for its proteasomal degradation (37). Previously, OTUD6B was reported to cleave the K48-linked ubiquitin chain and stabilize pVHL protein levels [24]. We found that OTUD6B also hydrolyzes K11 and K33 ubiquitin chains, which is a novel function of OTUD6B as a deubiquitinase.

Linear ubiquitination of IRF3 at Lys 193 and Lys 313 or 315 has been reported to activate the RLR-induced apoptosis pathway in RNA-virus-infected cells (46), which could also play an antiviral role. Notably, we found that K33-linked polyubiquitination at the Lys315 residue of IRF3 was cleaved by OTUD6B for IRF3 transcriptional activation. It is possible that different ubiquitination types at Lys315 of IRF3 compete with each other to contribute to the switch between transcriptional activation or apoptosis induction. Moreover, we noticed that the mutation of Lys315 did not completely block the IRF3 K33-linked ubiquitination level, which suggests that the Lys315 residue of IRF3 could not be the only site for K33-linked ubiquitination. The functions of other Lys residues linked to K33-ubiquitination need to be further elucidated. Notably, IRF3 has 14 lysine sites, and the effect of ubiquitin modifications at some of these sites has been reported. The K48-linked polyubiquitin chain at Lys70/Lys87 of IRF3 added by E3 ligase TRIM26 promotes IRF3 degradation after nuclear translocation (14). In addition, K48-linked polyubiquitination at Lys313 of IRF3 mediated by E3 ligase MID1 reduces IRF3 protein stability (11). However, evidence has shown that a K27-linked polyubiquitin chain exists at Lys313. The cleavage of this site polyubiquitin chain by the deubiquitinase PSMD14/POH1 prevents IRF3 autophagy degradation, thus maintaining the basal level of type I IFN activation (18). These studies suggest that IRF3 lysine at different sites can be modified using multiple types of polyubiquitin chains, and its polyubiquitination level is tightly controlled by E3 ligase and deubiquitinase. In our study, we found that the IRF3 Lys315 K33-linked polyubiquitin is cleaved by human OTUD6B and strongly associated with IRF3 stability. However, we cannot exclude the other E3 ligase such as c-Cbl- and MID1-mediated K48-linked polyubiquitination for IRF3 degradation (11, 47).

In summary, our study revealed that OTUD6B is a positive regulator of IRF3 protein expression. OTUD6B is able to interact with IRF3 and regulates IRF3 protein stability by mediating K33-linked deubiquitination modifications at Lys315. These findings reveal a novel biological function of OTUD6B. Furthermore, we demonstrated that OTUD6B can promote type I IFN production and cellular antiviral responses *in vivo*, which may provide a potential target for enhancing type I IFN antiviral therapy in clinical settings.

## MATERIALS AND METHODS

### Cells, mice, virus, and reagents

293T, HT1080, HEP2, Vero, and U3A cells were reserved by the laboratory. U3A was a STAT1-deficient cell line (14). 293T IRF3 KO cells were kindly provided by Prof. Fangfang Zhou from Soochow University as previously described (48). All cells were cultured in DMEM (HyClone) supplemented with 10% FBS, 100 U/mL penicillin, and 100 µg/mL streptomycin at 37°C under 5% CO2.

Six- to eight-week C57BL/6 mice were purchased from the Laboratory Animal Center of Soochow University. Mice were bred and housed in the animal facility of the Soochow University under specific pathogen-free conditions. All protocols and procedures for mice study have been approved by the Committees of the Scientific Investigation Board of Soochow University.

VSV, VSV-GFP, or HSV-1 was obtained and reserved by the laboratory. Sendai virus (SeV), H1N1 was a gift from Prof. Hui Zheng (Soochow University, Suzhou, China). RSV was a gift from Prof. Jinping Zhang (Soochow University, Suzhou, China).

Recombinant human IFNα was purchased from PBL InterferonSource (Waltham, MA, USA). IFNα was used at the concentration of 1,000 IU/mL unless stated otherwise. Cycloheximide, chloroquine (CQ), and MG132 were purchased from Sigma-Aldrich (St. Louis, MO, USA).

### Generation of human OTUs knockout library and screening

To create gene-targeted alleles encoding OTUs family in 293T cells, three CRISPR guide RNA (gRNA) sequences were chosen for each OTU based on their specificity scores (https://portals.broadinstitute.org/gppx/crispick/public) (Table 1). The DNA oligos encoding small gRNAs were cloned into the vector lentiCRISPRv2 (Addgene, catalog#98290) and prepared for lentiviral packaging, individually. Briefly, 5 × 10^6^ 293T cells seeded in 10 cm dish were transfected with 10 µg OTUs sgRNA plasmid or the empty vector along with 5 µg packaging vectors psPAX2 and 3 µg pVSVG. The medium was changed 6 h post transfection. Forty-eight hours later, supernatants were harvested to infect 293T cells followed by various analyses.

**TABLE 1:** List of all sequences for sgRNA

Name	Sequence
*OTUB1 Target sequence1*	5′-ggcctatgatgaagccatca-3′
*OTUB1 Target sequence2*	5′-ggagctctcggtcctataca-3′
*OTUB1 Target sequence3*	5′-gcaggaccgaattcagcaag-3′
*OTUB2 Target sequence1*	5′-ctgaaaacaggatttaccgg-3′
*OTUB2 Target sequence2*	5′-gatttaccggaggaaaatcg-3′
*OTUB2 Target sequence3*	5′-ttcgggaccatcctgaaaac-3′
*OTUD1 Target sequence1*	5′-accggctccgccgcactact-3′
*OTUD1 Target sequence2*	5′-ctcagtcggaagttccgatc-3′
*OTUD1 Target sequence3*	5′-ctcgtctctgggatccgacc-3′
*OTUD3 Target sequence1*	5′-taaggccatgtcccgaaagc-3′
*OTUD3 Target sequence2*	5′-ggccatgtcccgaaagcagg-3′
*OTUD3 Target sequence3*	5′-gcaggcggcgaagagccggc-3′
*OTUD4 Target sequence1*	5′-agagacaccccgttaatttg-3′
*OTUD4 Target sequence2*	5′-cgcaaagcagcgtcccactc-3′
*OTUD4 Target sequence3*	5′-tcccactccggcgttacaat-3′
*OTUD5 Target sequence1*	5′-agtggactagccggtccccg-3′
*OTUD5 Target sequence2*	5′- aagcagtcagttctcggcag −3’
*OTUD5 Target sequence3*	5′-agttctcggcaggggccgac-3′
*OTUD6A Target sequence1*	5′-acatcgtgcgcaccacggca-3′
*OTUD6A Target sequence2*	5′-actcgcccaccttgatcatc-3′
*OTUD6A Target sequence3*	5′-atgaaagcgatcccggccga-3′
*OTUD6B Target sequence1*	5′-aggtgcctactagccggtgc-3′
*OTUD6B Target sequence2*	5′-atccggtgccgccttgaagg-3′
*OTUD6B Target sequence3*	5′-attgaccgaagagcttgatg-3′
*OTUD7A Target sequence1*	5′-aggcaggacgacattgccca-3′
*OTUD7A Target sequence2*	5′-cagtcagaaagcctctccag-3′
*OTUD7A Target sequence3*	5′-catgtgttcaatgaagggcg-3′
*OTUD7B Target sequence1*	5′-ccgcagctgttgctccgca-3′
*OTUD7B Target sequence2*	5′-attcctcggcagtgaccccg-3′
*OTUD7B Target sequence3*	5′-ctgttgctccgcacgggatc-3′
*VCPIP1 Target sequence1*	5′-gagcttattcgaatagctcc-3′
*VCPIP1 Target sequence2*	5′-ggagtagtaacaatgagaga-3′
*VCPIP1 Target sequence3*	5′-tagtaacaatgagagacggc-3′
*YOD1 Target sequence1*	5′-agtgtgtactatgtcgtcga-3′
*YOD1 Target sequence2*	5′-ctgcaacggatgatacagcc-3′
*YOD1 Target sequence3*	5′-gcagtcgtcttgaagaacca-3′

CRISPR-Cas9 genomic editing for gene deletion was used as previously reported (49). The library contains 13 lentiviruses for human OTUs family knockout. 293T cells were infected with either control sgRNA or sgRNA lentivirus against a single human OUT for 48 h. The cells were then infected with VSV-GFP (MOI = 1.0) for 12 h and analyzed by flowcytometry for viral infection.

### Plasmids and transfection

Plasmids for RIG-FL, RIG-IN, IRF3/5D, MAVS, TBK1, and IRF3 WT were saved by the laboratory. Luciferase reporter plasmids (IFN-β-Luc and IFN-stimulated response elements [ISRE]-Luc) and other plasmids including HA-Ub, HA-Ub-K6, HA-Ub-K11, HA-Ub-K33R, HA-Ub-K27, HA-Ub-K29, HA-Ub-K33, HA-Ub-K48, HA-Ub-K63, and Flag-IRF3 were gifts from Professor Hui Zheng (Soochow University, Suzhou, China).

The human OTUD6B cDNA was PCR amplified from 293T cells with the following primer pair sequence as shown in Table 2. The amplified fragment was cloned into pCMV-HA vector using EcoRI and XhoI restriction enzyme sites and named HA-OTUD6B. And, we also cloned the human OTUD6B-amplified fragment into pCMV-Myc vector using EcoRI and XhoI restriction enzyme sites and named myc-OTUD6B. We also used the Site-Directed Mutagenesis Kit (Takara Bio, Tokyo, Japan) to generate C57S-mutated OTUD6B (HA-OTUD6B-C57S) with the following primers OTUD6B CS-F and OTUD6B CS-R; IRF3-WT is also mutated to IRF3-K313R, IRF3-K315R, IRF3-K360R, IRF3-K366R, or IRF3-K409R with the following primers in Table 2. The mutated sites were underlined in the primer sequence. Using IRF3-WT as a template, we constructed IRF3 ∆C and IRF3 ∆N using the primers in Table 2. The OTUD6B-knockdown shRNA was constructed by inserting the OTUD6B shRNA fragment into empty plasmid pLL3.7. The three OTUD6B shRNA target sequences were obtained from the Sigma Mission Library and were shown in Table 2. All plasmids were confirmed by DNA sequencing. The OTUD6B siRNA (siOTUD6B) and control scramble siRNA were purchased from RiboBio (siG000051633 and siN0000001).

**TABLE 2:** Primer list for plasmid constructions

Primer	Sequence
For overexpression plasmid construction	
HA-hOTUD6B -F(pCMV-HA)	5′-gcccg **aattc** ggatgatatctaaggaaaagaaagctgcattg-3′
HA-hOTUD6B -R(pCMV-HA)	5′-ccg **ctcgag** cttagctgcaattttcagtaactatgtttacc-3′
myc-hOTUD6B -F(pCMV-myc)	5′-gccc **gaattc** ggatgatatctaaggaaaagaaagctgcattg-3′
myc-hOTUD6B -R(pCMV-myc)	5’-ccg **ctcgag** cagctgcaattttcagtaactatgtttacc-3’
For point mutation plasmid construction	
OTUD6B CS-F	5′-agattccatctgatggccac **agt** atgtataa-3′
OTUD6B CS-R	5′-tgtggccatcagatggaatctgtttaatttc-3′
IRF3-K313R-F	5′-atggcgaggtcccc **agg** gacaaggaa-3′
IRF3-K313R-R	5′-ctggggacctcgccatcaggcccatg-3′
IRF3-K315R-F	5′-aggtccccaaggac **agg** gaaggaggc-3′
IRF3-K315R-R	5′-ctgtccttggggacctcgccatcagg-3′
IRF3-K360R-F	5′-accagccgtggacc **agg** aggctcgtg-3′
IRF3-K360R-R	5′-ctggtccacggctggtcctggggcca-3′
IRF3-K366R-F	5′-ggctcgtgatggtc **agg** gttgtgccc-3′
IRF3-K366R-R	5′-ctgaccatcacgagcctcttggtcca-3′
IRF3-K409R-F	5′-cctccgaccagtac **agg** gcctacctg-3′
IRF3-K409R-R	5′-ctgtactggtcggaggtgagggagag-3′
For truncation plasmid construction	
IRF3 ΔC-F	5′- gccgaattcaatgggaaccccaaagccacg −3′
IRF3 ΔC-R	5′- gcctctagatcaccccggcaccaacagcc-3′
IRF3 ΔN-F	5′- gccgaattcagaagagtgggagttcgaggtgacag-3′
IRF3 ΔN-R	5′- gcctctagatcagctctccccagggccc-3′
For shRNA knockdown plasmid construction	
OTUD6B Sh1-F(pLL3.7)	5′-tgcaaagctactaacaggtgttttcaagagaaacacctgttagtagctttgcttttttc-3′
OTUD6B Sh1-R(pLL3.7)	5′-tcgagaaaaaagcaaagctactaacaggtgtttctcttgaaaacacctgttagtagctttgca-3′
OTUD6B Sh2-F(pLL3.7)	5′-tcgagaagaacggatagctgaattcaagagattcagctatccgttcttctcgttttttc-3′
OTUD6B Sh2-R(pLL3.7)	5′-tcgagaaaaaacgagaagaacggatagctgaatctcttgaattcagctatccgttcttctcg a-3′
OTUD6B Sh3-F(pLL3.7)	5′-tcgatgagactaatgcagtgaattcaagagattcactgcattagtctcatcgttttttc-3′
OTUD6B Sh3-R(pLL3.7)	5′-tcgagaaaaaacgatgagactaatgcagtgaatctcttgaattcactgcattagtctcatcga-3′

All transfections were carried out using Longtrans (UcallM Biotechnology, Wuxi, China) for DNA plasmids or Lipofectamine RNAiMAX (Invitrogen, Carlsbad, CA, USA) for siRNA according to the manufacturer’s instructions.

### Generation of OTUD6B stable knockdown cell line 293T sh-OTUD6B

To downregulate OTUD6B gene expression in 293T cells, three shRNA sequences with high scores targeting human-derived OTUD6B were selected from the Sigma-Aldrich website (Table 2). The DNA oligos encoding shRNAs were cloned into the vector pLL3.7 (Addgene, catalog #11795), and the plasmids validated to have the highest downregulation efficiency were prepared for lentiviral packaging.

293T cells were co-transfected with OTUD6B knockdown shRNA plasmid and the packaging vector, psPAX2 and pVSVG, using Lipofectamine3000 (Invitrogen). The cell culture supernatant was harvested 48 and 72 h post transfection and filtered through a 0.45-µm pore size filter. 293T cells were infected with viral supernatant at 37°C for 4 h and then cultured in fresh medium for 72 h. The GFP-positive cells were sorted by BD FACS Aria II flow cytometry (Becton Dickinson), and 293T-Vector cells were generated by infection with pLL3.7-packaged lentivirus as a control. The downregulation of OTUD6B in knockdown cells was verified by western blotting.

### Viral infection

To assess the antiviral ability of OTUD6B, control siRNA or siOTUD6B was transfected into cells. After 48 h post transfection, cells were infected with VSV-GFP, RSV, SEV, H1N1, and HSV-1 at MOI = 1.0 for 12 h. Cells were then collected, and viral infection was analyzed by flow cytometry, western blot, real-time PCR, and fluorescence microscope. To identify whether OTUD6B regulates IFN downstream-related signaling, cells were transfected with HA-OTUD6B or pCMV-HA. Forty-eight hours post transfection, cells were treated with 30 IU/mL IFN-α for 15 h. The expression of ISGs level was analyzed by real-time PCR.

For *in vivo* mouse infection, polyplexes were prepared at a nitrogen/DNA phosphate ratio = 7. The polymer and 50 µg of DNA were both diluted in 5% dextrose to a volume of 100 µL, respectively. DNA and polymer were then mixed, whirled for 30 s, and incubated for 30 min in a 200 µL volume after mixing. Each mouse was i.v. injected with 50 µg of PEI-packaged pCMV-HA plasmid or human HA-OTUD6B overexpression plasmid. On day 2 post injection, the lung tissues were removed from sacrificed mice, and OTUD6B expression was detected by western blot. For other parallel groups, the mice were intranasally infected with 1 × 10^8^ PFUs VSV-GFP on day 1 post injection. The mRNA levels of IFN-β or ISGs in mice lung tissues were analyzed by real-time PCR 3 days post infection. Meanwhile, VSV G protein level in infected mice lung tissues was detected by western blot, and serum viral titer was detected by quantitative PCR. For survival rate analysis, the survival of each group containing eight infected mice was monitored until day 10 post infection.

### Western blot

Cells were harvested using lysis buffer containing 150 mM NaCl, 20 mM Tris–HCl (pH 7.4), 1% NP-40, 0.5 mM EDTA, PMSF (50 mg/mL), and protease inhibitors (NCM, Suzhou, China). Equivalent protein aliquots were subjected to SDS-PAGE and transferred to PVDF membranes. Membranes were then blocked with 5% fat-free milk or 5% bovine serum albumin (BSA) for 2 h at room temperature and then probed with the primary antibody, followed by the corresponding HRP-conjugated goat anti-mouse or goat antirabbit secondary antibodies (SouthernBiotech, Birmingham, AL, USA). The following Abs were used: antibodies against OTUD6B (1:2,000; HPA024046; Sigma-Aldrich), IRF3 antibody generated from rabbit (1:1,000; 11904S; CST, Danvers, MA, USA), IRF3 antibody generated from mouse (1:1,000; ab50772; Abcam), VSV-G (1:5,000; sc-66180; Santa Cruz Biotechnology), HA (1:2,000; 3724S; CST), ubiquitin (1:1,000; sc-8017; Santa Cruz Biotechnology), Flag (1:2,500; F7425; F1804; Sigma-Aldrich), Myc (1:2,000; 2272S; CST), and GAPDH (1:20,000; G9545; Sigma-Aldrich). The band intensities were quantified by ImageJ software (Media Cybernetics, Silver Spring, MD, USA).

### Immunoprecipitation analysis of OTUD6B and IRF3 interaction

293T cells were transfected with HA-OTUD6B plasmid for 36 h. The cells were then washed with pre-cooled 1× PBS and lysed in western and IP lysis buffer (Beyotime) containing 100 µM PMSF (Beyotime). Cell lysates were collected and incubated on a shaker with 25 µL Anti-HA Magnetic beads (Bimake) at 4°C overnight. The beads were eluted with 1× loading buffer after washing five times with lysis buffer and boiled for 15 min. The proteins were analyzed by western blot analysis with anti-HA and anti-IRF3 antibody.

For detecting endogenous OTUD6B and IRF3 interaction, 293T cells infected with VSV were washed with pre-cooled 1× PBS and lysed in IP lysis buffer containing 100 µM PMSF. Cell lysates were incubated with anti-IRF3 antibody at 4°C overnight and then immunoprecipitated with protein A agarose at room temperature for 3 h. The beads were eluted with 1× loading buffer after washing five times with lysis buffer and boiled for 15 min. The proteins were analyzed by western blot analysis using anti-OTUD6B antibody.

### Reporter gene assay

Reporter gene assay for detecting the effect of OTUD6B on IFN-β production, 1 × 10^5^ 293T cells were seeded in a 24-well plate each well and cells were transfected with empty vector or OTUD6B expression plasmid (800 ng), IFN-β promoter firefly luciferase reporter plasmid (200 ng), and TK-Renilla luciferase reporter plasmid (20 ng), together with empty vector or an expression plasmid (200 ng) for the RIG-I amino-terminal “2CARD” domain (RIG-IN), MAVS, TBK1, or IRF3/5D. Forty-eight hours post transfection, the luciferase activities were measured using the Dual-Luciferase Reporter Assay System (Promega, Madison, WI, USA; E1910).

### Immunofluorescence assay

For detecting variation of IRF3 amount in the nucleus, 293T cells were transfected with HA-Vector or HA-OTUD6B plasmid. Thirty-six hours post transfection, the cells were infected with SeV for 12 h. The cells were then washed thrice with 1× PBS, fixed with 4% paraformaldehyde, and permeabilized with 0.05% Triton X-100. The cells were then blocked with 3% bovine serum albumin (Sigma) and incubated with anti-IRF3 mouse antibody and anti-HA rabbit antibody at 4°C overnight. The next day, cells were washed with 1× PBS three times and incubated with DyLight 488-goat anti-mouse IgG (Jackson, 111-545-003) and DyLight 633-goat anti-rabbit IgG (Jackson, 111-605-144) for 30 min at room temperature, and the nuclei were stained with DAPI (Beyotime) for 15 min at room temperature. The pictures were acquired by confocal microscopy Nikon A1 (scale bars: 5 µM). The quantification of the mean fluorescence intensity of IRF3 in the nucleus is given automatically by the Image J software calculation.

### CHX chase assay

The half-life of total IRF3 was determined by CHX chase assay. For analysis of total IRF3 level, 293T cells were transfected with HA-OTUD6B or other indicated plasmids for 48 h. The cells were then treated with CHX (50 mg/mL) for 0, 6, and 12 h and lysed for western blot. The gray intensity of protein band was measured by using ImageJ software version 1.6.0_20. The relative amount of IRF3 was calibrated by the GAPDH. The IRF3 protein levels at A time points were calculated as (IRF3_A h/GAPDH_A h)/(IRF3_0 h/GAPDH_0 h).

### MG132 assay

A total of 1 × 10^6^ 293T cells seeded in a 60-mm dish were transfected with 1 µg HA-K33, together with 1.5 µg of Flag-IRF3 plasmid for 36 h. The cells were treated with MG132 (10 mM) or CQ (100 mM) for 6 and 12 h. Cell lysates were incubated with anti-Flag beads (Anti-Flag magnetic beads, Bimake) at 4°C overnight, and the K33- or K11-linked ubiquitination of IRF3 was analyzed by western blot.

### *In vivo* deubiquitination assay

293T cells were transfected with the indicated plasmids and treated with MG132 (5 mM) for 4 h before harvesting. Forty-eight hours after transfection, cells were washed with PBS and lysed in RIPA buffer (20 mM Tris-base, pH 7.4, 150 mM NaCl, 1% Triton, 0.5% sodium-deoxycholate, and 1% SDS) supplemented with protease inhibitors and 10 mM *N*-ethylmaleimide. The supernatant was incubated with anti-Flag beads overnight at 4°C. After extensive washing, bound proteins were eluted with 5× loading buffer and separated by SDS-PAGE, followed by western blot analysis.

### RNA isolation and real-time PCR

Total RNA was extracted from cells using TRIzol (Takara), and cDNA synthesis was performed using random primers with 500 ng of total RNA. Real-time PCR was performed using an SYBR Green PCR Master Mix (Applied Biosystems, Waltham, MA, USA) with specific primers (Table 3) and normalized with the human GAPDH gene. Gene expression was analyzed using the 2^−△△CT^ method (50, 51). VSVN-F and VSVN-R primers were used to amplify partial VSV viral N gene fragments, which were cloned into pEASY-Blunt vector as quantitative PCR standard. The VSV viral titer was measured using quantitative PCR, and the probe was 5′-FAM-atccgagccattcgaccaca-Eclipse-3′.

**TABLE 3:** List of primers for real-time PCR analysis

Target gene	Gene ID	Sequence
GAPDH-F	2597	5′-agggctgcttttaactctggt-3′
GAPDH-R		5′-ccccacttgattttggaggga-3′
hIFN-b-F	3456	5′-cattacctgaaggccaagga-3′
hIFN-b-R		5′-cagcatctgctggttgaaga-3′
hIFIT1-F	3434	5′-cacaagccattttctttgct-3′
hIFIT1-R		5′-acttggctgcatatcgaaag-3′
hISG54-F	3433	5′-cacctctggactggcaatagc-3′
hISG54-R		5′-gtcaggattcagccgaatgg-3′
mIFN-b-F	15977	5′-tccgagcagagatcttcaggaa-3′
mIFN-b-R		5′-tgcaaccaccactcattctgag-3′
mISG54-F	15958	5′-agaaccaaaacgagagagtgaag-3′
mISG54-R		5′-tccagacggtagttcgcaatg-3′
mIFIT1-F	15957	5′-atcgcgtagacaaagctcttc-3′
mIFIT1-R		5′-gtttcgggatgtcctcagttg-3′
mGAPDH-F	14433	5′-tggccttccgtgttcctac-3′
mGAPDH-R		5′-gagttgctgttgaagtcgca-3′
OTUB1-F	55611	5′-ctgacggcaactgtttctatcg-3′
OTUB1-R		5′-caggtccatgaacgtgttgtg-3′
OTUB2-F	78990	5′-ttgaggagcacaagttcagaaac-3′
OTUB2-R		5′-gcagaagtctttgatgtccatct-3′
OTUD1-F	220213	5′-ggggagtttatcatcgctgct-3′
OTUD1-R		5′-tgagccaactgagccaaatac-3′
YOD1-F	55432	5′-atgtttggccccgctaaagg-3′
YOD1-R		5′-cggtgatggcggcaatttg-3′
OTUD3-F	23252	5′-taaagcagcgggaagattttga-3′
OTUD3-R		5′-tgcgatgtgtaactccctcac-3′
OTUD4-F	54726	5′-tagcaatccatgtgtccagaga-3′
OTUD4-R		5′-aagggcactctaacttctttgac-3′
OTUD5-F	55593	5′-ggttgtgcgaaagcattgcat-3′
OTUD5-R		5′-acctccacaggacggttgt-3′
OTUD6A-F	139562	5′-gaagttccaagacgacagtagc-3′
OTUD6A-R		5′-caggtgctccgacatctca-3′
OTUD6B-F	51633	5′-gaagtcagaccgctgagtatatg-3′
OTUD6B-R		5′-gggaggagaatctgcctgtatta-3′
OTUD7A-F	161725	5′-cacgagctgtaaacggcttct-3′
OTUD7A-R		5′-gctttccgtaacaccaggtcc-3′
OTUD7B-F	56957	5′-gacagagagcctactcgcc-3′
OTUD7B-R		5′-cacagatgggcatttccagg-3′
VCPIP1-F	80124	5′-agtgtcaggcgcgtctatttt-3′
VCPIP1-R		5′-gagctaatatgggcgacaacaat-3′
VSV-F		5′-ctcggttcaagatccaggt-3′
VSV-R		5′-acggcgtacttccagatgg-3′
HSV-1-F		5′-cgcatcaagaccacctcctc-3′
HSV-1-R		5′-agcttgcgggcctcgtt-3′
H1N1-F		5′-ttctaaccgaggtcgaaacg-3′
H1N1-R		5′-acaaagcgtctacgctgcag-3′
RSV-F		5′-aagggatttttgcaggattgttt-3′
RSV-R		5′-ctccccaccgtagcattacttg-3′
SeV-F		5′-gatgacgatgccgcagcagtag-3′
SeV-R		5′-cctccgatgtcagttggttcactc-3′
VSVN-F		5′-cagcaggtttgttgtacgcttat-3′
VSVN-R		5′-gagtcggttttctgttttgatctt-3′

### ELISA assay

ELISA assays were performed in 96-well ELISA plates using human IFN-β ELISA kits (RD system) and mouse IFN-β ELISA Kit (FCMACS; FMS-ELM067) according to the manufacturer’s instructions.

### Flow cytometry analysis

293T cells infected with VSV-GFP (MOI = 1.0) were subjected to analysis by flow cytometry. For flow cytometry analysis, cells were collected with cold PBS and were acquired in a FACS Canto II (BD Biosciences, San Jose, CA, USA) equipped with a 488-nm argon laser. FACS data were analyzed with FlowJo software (FlowJo, Ashland, OR, USA).

### Fluorescence microscopy

293T cells were transfected with siOTUD6B (100 nM) for downregulation or HA-OTUD6B (1.0 or 1.5 µg) for up-regulation. After 36 h post transfection, cells were infected with VSV-GFP (MOI = 1.0) for 12 h, and the viral infection was measured by fluorescence microscope (Nikon Eclipse TS100). A barely perceptible shadow in the lower right corner of all the fluorescence images was rechecked and found to be the result of the filter being misaligned.

### Statistical analysis

Comparison between different groups was analyzed by two-tailed Student *t*-test. Data were shown as the mean ± SD. The *P* value <0.05 was considered statistically significant. Kaplan–Meier survival curves were generated and analyzed for mice survival study performed in GraphPad Prism 9.0 (GraphPad Software, San Diego, USA).

## SUPPLEMENTAL MATERIAL

Fig. S1 mbio.00332-23-s0001.tif The protein expressions of each group in IFN luciferase reporter assay.

Fig. S2 mbio.00332-23-s0002.tif IRF3 underwent K33-linked ubiquitination.

Fig. S3 mbio.00332-23-s0003.tif K33-linked IRF3 ubiquitination was responsible for degradation.

Fig. S4 mbio.00332-23-s0004.tif K315 site in IRF3 was responsible for protein degradation.

ASM does not own the copyrights to Supplemental Material that may be linked to, or accessed through, an article. The authors have granted ASM a non-exclusive, world-wide license to publish the Supplemental Material files. Please contact the corresponding author directly for reuse.
