# Supplementary figures and images for: Human OTUD6B positively regulates type I IFN antiviral innate immune responses by deubiquitinating and stabilizing IRF3
Source: mBio. 2023 Aug 31;14(5):e00332-23. doi: 10.1128/mbio.00332-23 (PMC10653906; doi:10.1128/mbio.00332-23)

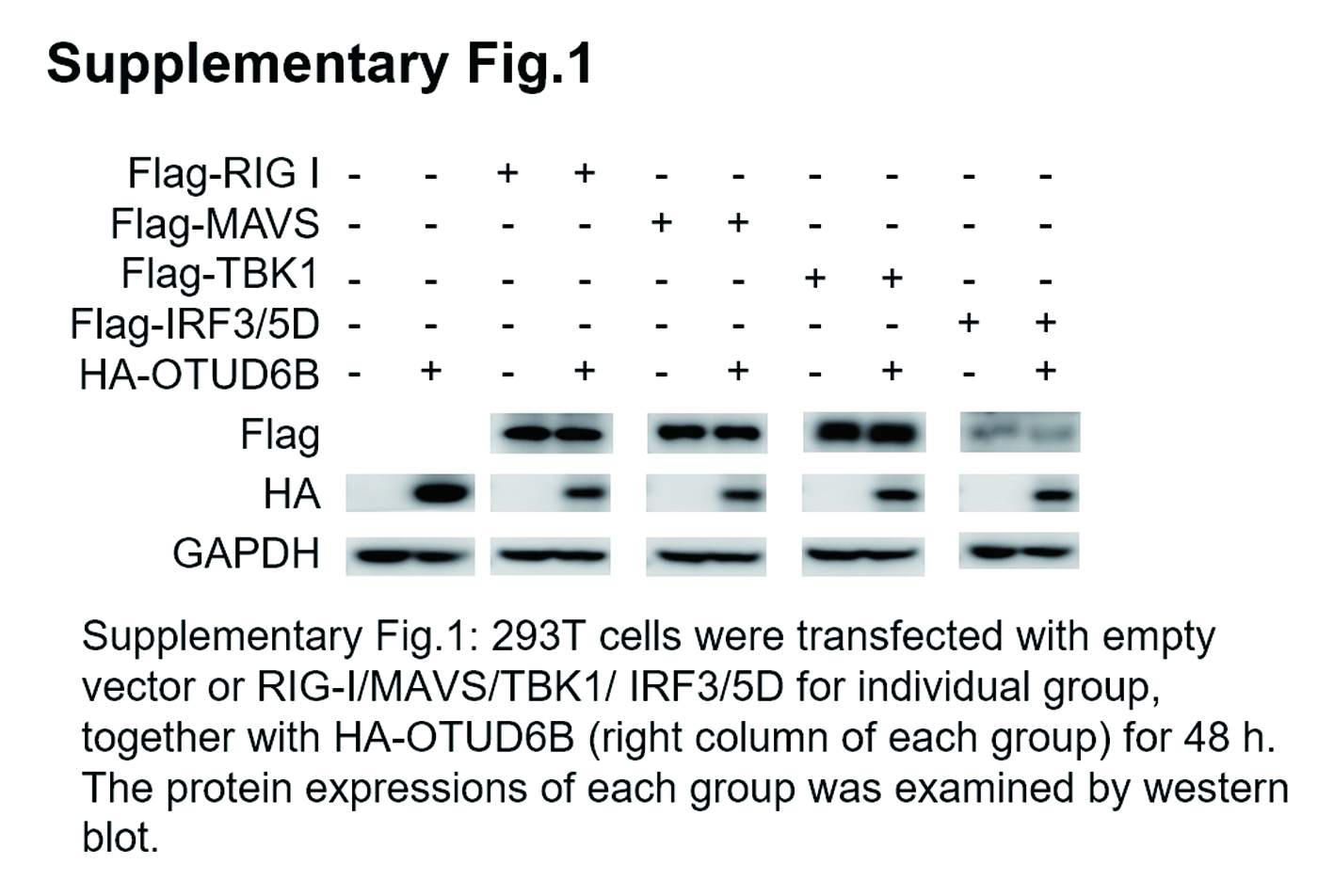

Supplement: Fig. S1 — The protein expressions of each group in IFN luciferase reporter assay. [file mbio.00332-23-s0001.tif]

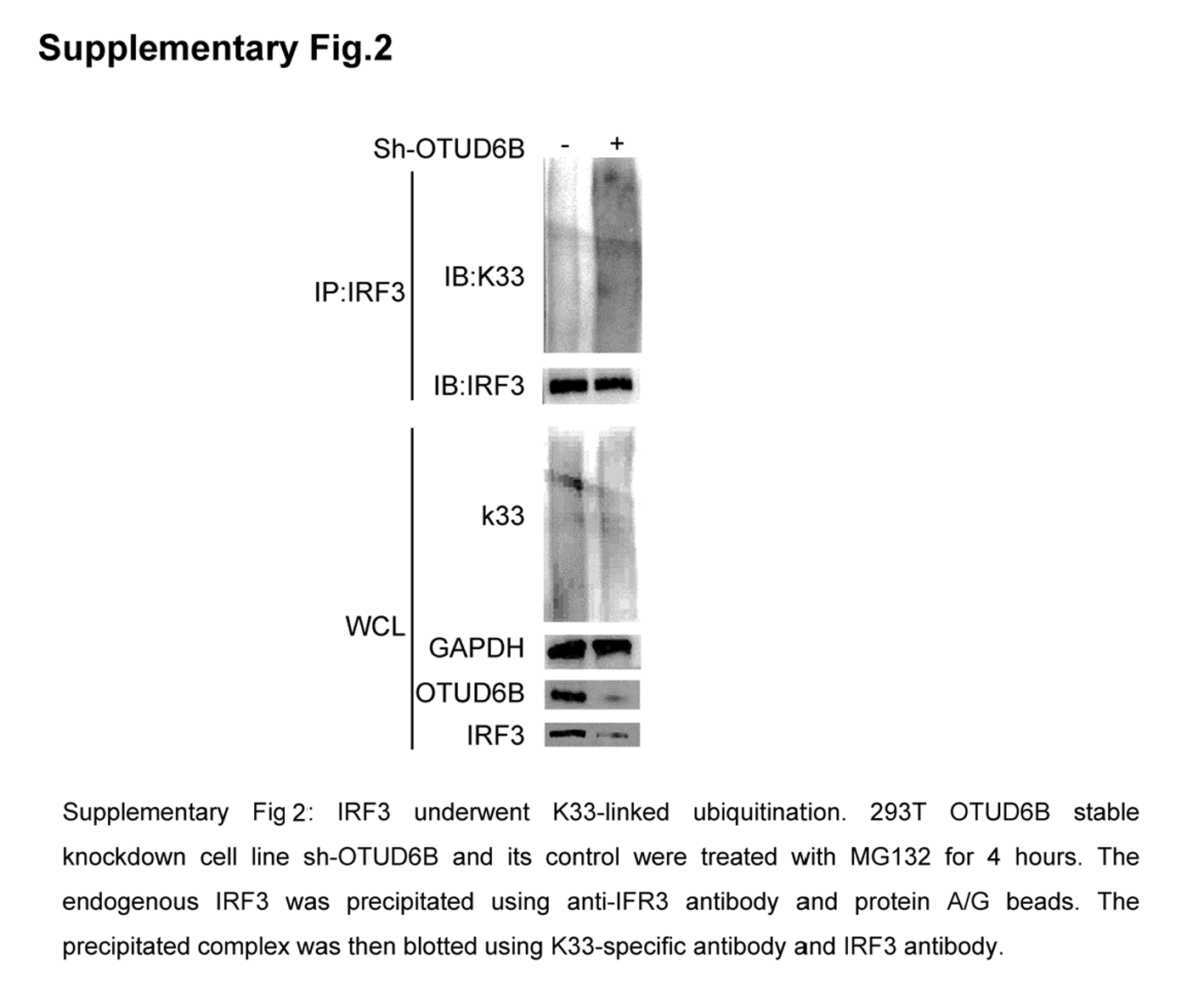

Supplement: Fig. S2 — IRF3 underwent K33-linked ubiquitination. [file mbio.00332-23-s0002.tif]

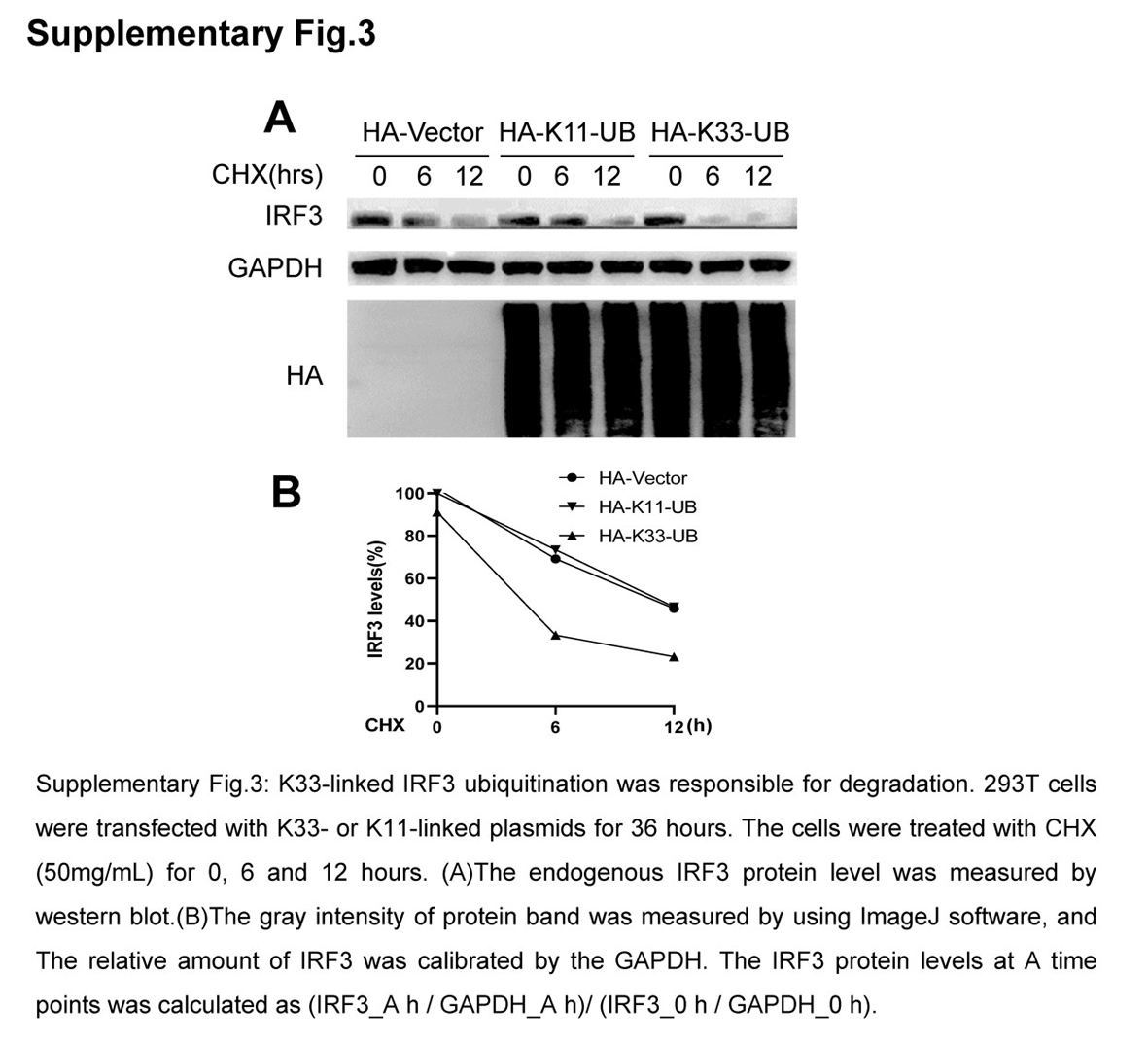

Supplement: Fig. S3 — K33-linked IRF3 ubiquitination was responsible for degradation. [file mbio.00332-23-s0003.tif]

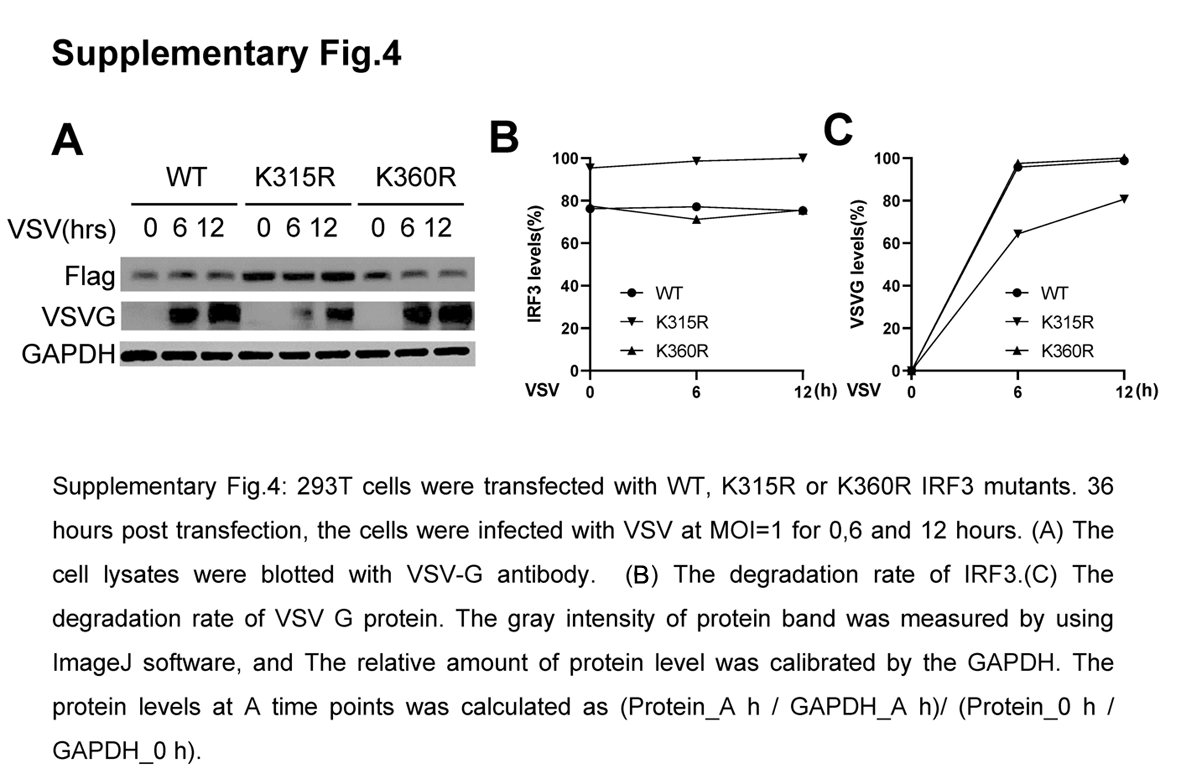

Supplement: Fig. S4 — K315 site in IRF3 was responsible for protein degradation. [file mbio.00332-23-s0004.tif]
